# Impact of Concurrent aerobic–anaerobic Methanotrophy on Methane Emission from Marine Sediments in Gas Hydrate Area

**DOI:** 10.1021/acs.est.3c09484

**Published:** 2024-03-06

**Authors:** Yusuke Miyajima, Tomo Aoyagi, Hideyoshi Yoshioka, Tomoyuki Hori, Hiroshi A. Takahashi, Minako Tanaka, Ayumi Tsukasaki, Shusaku Goto, Masahiro Suzumura

**Affiliations:** † Research Institute for Geo-Resources and Environment, Geological Survey of Japan, National Institute of Advanced Industrial Science and Technology (AIST), Central 7, 1-1-1 Higashi, Tsukuba, Ibaraki 305-8567, Japan; ‡ Environmental Management Research Institute, National Institute of Advanced Industrial Science and Technology (AIST), 16-1 Onogawa, Tsukuba, Ibaraki 305-8569, Japan; § Research Institute of Earthquake and Volcano Geology, Geological Survey of Japan, National Institute of Advanced Industrial Science and Technology (AIST), Central 7, 1-1-1 Higashi, Tsukuba, Ibaraki 305-8567, Japan; ∥ KANSO TECHNOS Co., Ltd., 14 Kanda Higashimatsushita-cho, Chiyoda-ku, Tokyo 101-0042, Japan

**Keywords:** gas hydrate, lipid, marine sediment, methane flux, methane oxidation, 16S rRNA, stable isotope probing

## Abstract

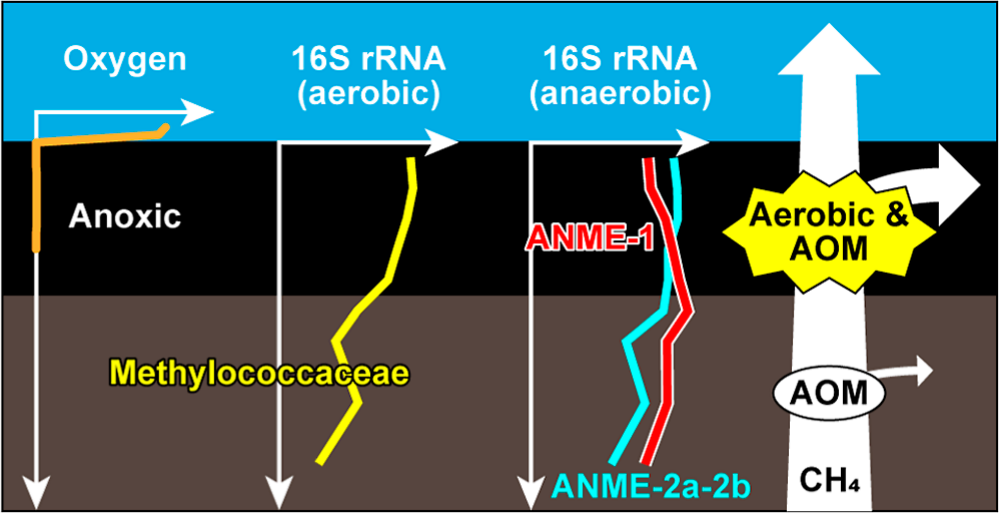

Microbial methane oxidation has a significant impact on the methane flux from marine gas hydrate areas. However, the environmental fate of methane remains poorly constrained. We quantified the relative contributions of aerobic and anaerobic methanotrophs to methane consumption in sediments of the gas hydrate-bearing Sakata Knoll, Japan, by *in situ* geochemical and microbiological analyses coupled with ^13^C-tracer incubation experiments. The anaerobic ANME-1 and ANME-2 species contributed to the oxidation of 33.2 and 1.4% methane fluxes at 0–10 and 10–22 cm below the seafloor (bsf), respectively. Although the aerobic Methylococcaceae species consumed only 0.9% methane flux in the oxygen depleted 0.0–0.5 cmbsf zone, their metabolic activity was sustained down to 6 cmbsf (based on rRNA and lipid biosyntheses), increasing their contribution to 10.3%. Our study emphasizes that the co-occurrence of aerobic and anaerobic methanotrophy at the redox transition zone is an important determinant of methane flux.

## Abstract 2

Geochemistry coupled with stable isotope probing of microbial cell components reveals that co-occurrence of aerobic and anaerobic methanotrophs is vital for accurate assessment of methane consumption in low flux marine sediments.

## Introduction

Microorganisms play a major role in regulating release of methane from marine sediments at the seafloor to the ocean and atmosphere. Methane is a potent greenhouse gas but also an important energy reserve, vast quantities of which are stored as free- or dissolved-gas and gas hydrates in marine sediments.^1,2^ The atmospheric methane flux from the sediments in gas hydrate areas is no larger than several percent of the current global flux.^3,4^ The oceanic methane flux to the atmosphere remains small, largely due to microbial oxidation of methane in the sediments and water column, referred to as the “microbial methane filter” or “benthic filter for methane”.^5−7^ Methane oxidation occurs via both aerobic and anaerobic processes, as indicated by eqs 1 and 2
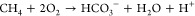
1
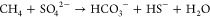
2

The proportion of the methane consumed, i.e., the efficiency of the microbial methane filter, was found to vary with the fluid flow rate and redox state at the seafloor.^7−9^ According to a previous study,^7^ 40–100% (mostly >70%) of methane is consumed at methane seeps with low fluid flow rates, resulting in methane effluxes of <50 mmol m^–2^ d^–1^. On the other hand, less than 30% of methane is consumed at seeps with higher fluid flow rates, which produce methane effluxes of >100 mmol m^–2^ d^–1^. The redox transition zones would be expanded more broadly in low flux regimes than in settings with higher fluxes of reducing fluid and gas, greatly influencing the relative contribution of aerobic to anaerobic methane oxidation. The filter efficiency was based on the anaerobic oxidation of methane (AOM) rates determined by sediment incubation, while the aerobic oxidation rates were estimated by the oxygen uptake rates at the seafloor. Oxygen uptake rates are sensitive to microbial respiration processes such as the oxidation of sulfides and organic materials. Therefore, little is known about the extent to which each oxidation path contributes to methane consumption, and the environmental fate of methane remains poorly constrained. The incubation-based determination of both aerobic and anaerobic oxidation rates is important to accurately assess their impacts on methane fluxes from marine sediments of gas hydrate-bearing areas.

Molecular analysis of microbial 16S rRNA genes and cell-membrane lipids in methane-rich marine sediments has revealed the possible involvement of the aerobic Gammaproteobacteria^5,10,11^ and anaerobic methanotrophs (ANMEs)^5,12,13^ in methane oxidation. However, these persistent biomarkers are derived from not only living but also fossil cells, which complicates the accurate identification of candidate methanotrophs. Targeting delicate but more physiologically relevant molecules such as 16S rRNA could effectively solve this issue. Further, in sediment incubation amended with ^13^C-labeled methane, stable isotope probing (SIP) can directly determine the contributions of methanotrophs to methane oxidation activity. Recent advances in rRNA-SIP enable the identification of metabolically active species with high-sensitivity,^14,15^ while lipid-SIP can be employed to confirm their molecular signatures.^16,17^ A combination of *in situ* rRNA surveys and SIP incubation experiments should provide more accurate information on the activity and distribution of key methanotrophic species and elucidate the processes of methane consumption in marine sediments.

The Sakata Knoll sediments in the northeastern Japan Sea (a gas hydrate-bearing area) have shown a low efflux of methane,^18,19^ serving as a suitable model ecosystem to pursue the environmental fate of methane. In this study, we investigated the geochemical parameters, including methane efflux rates and redox conditions and living microbial communities in the surface sediments of Sakata Knoll by *in situ* experiments. We then examined the methane oxidation rates and active methanotroph species by ^13^C-tracer incubation experiments, followed by rRNA- and lipid-based SIP. Combining these data, we determined the *in situ* distribution of the active species and quantified the relative contributions of the aerobic and anaerobic methanotrophs to methane consumption in the marine sediments of this gas hydrate area (Figure S1).

## Materials and Methods

### Sediment Sampling and Geochemical Analysis

Sakata Knoll is located ∼525 m below sea level off Sakata City, Yamagata Prefecture (Figure S2A,B). The knoll is an anticlinal structure adjacent on the east side to a reverse fault with a northeast-southwest trend. The presence of gas hydrates below the seafloor was indicated by high resistivity zones in the Logging While Drilling (LWD) data.^18^ A seismic survey showed acoustic blanking features likely associated with gas-bearing fluids.^19^ Abundant white and gray-colored microbial mats were observed on the seafloor sediments,^20^ indicating the reducing fluid flow and anaerobic methane oxidation activity.^21^

Push core samples were collected at two sites of the Sakata Knoll in July 2020 using an ROV Hakuyo 3000 during the SS20-1 cruise of the R/V Shinseimaru (Fukada Salvage and Marine Works Co., Ltd., Osaka, Japan) (Figure S2 and Table S1). The LB1-PC site was located close to a central depression of the knoll and characterized by a surface covered with a white and gray microbial mat of 4–5 m diameter. Surface sediment samples were taken from outside and inside the microbial mat (LB1-PC-OBM and -IBM, respectively) and a reference site (REF-PC) located ∼1.8 km northwest of the LB1-PC site using an MBARI polycarbonate push core (40 cm length, 7 cm inner diameter). Recovered sediments were immediately separated into slices of 2 cm length and stored at either room temperature, 4 °C, or −80 °C depending on the required analytical procedures (Table S1). Pore waters were squeezed from whole-round core samples as previously described.^20^ Concentrations of headspace methane, pore-water sulfate, sulfide, and dissolved inorganic carbon (DIC) and stable carbon isotopic composition (in δ^13^C) of methane and DIC were measured as described in previous study^20^ and Section SB.1. Pore fluid flow rate was estimated by monitoring bottom-water and sediment temperatures (Sections SB.3 and SB.4). Microsensor analysis of dissolved oxygen (DO) concentration and oxidation–reduction potential (ORP) was carried out immediately after sample collection as described in Section SB.2.

### 16S rRNA Gene and Transcript Sequencing and Lipid Biomarker Analysis

Nucleic acids were extracted in triplicate from 0.4 to 0.7 g of the frozen wet sediments (0.2–0.5 g in dry weight) and 1 mL of incubation slurries using a direct lysis protocol involving bead beating.^22^ Total DNA and RNA were purified by digestion with RNase (Type II-A; Sigma-Aldrich, St. Louis, MO, USA) and DNase (RQ1; Promega, Madison, WI, USA), respectively. Quantitative polymerase chain reaction (qPCR) and reverse transcription (RT) qPCR of the DNA and RNA were carried out with the universal primer set 515F/806R^23^ targeting the 16S rRNA V4 region, as previously described.^24,25^ 16S rRNA copy numbers were quantified using standard curves generated from *Escherichia coli* gene fragments. For high-throughput sequencing, PCR and RT-PCR were performed as previously described.^26^ Both the 515F and 806R primer was modified to contain an adapter region, and the reverse primer was encoded with 12-bp barcodes.^23^ The thermal conditions were the same as previously described,^24,25^ except for the 25–30 and 22–25 cycles employed for PCR and RT-PCR. For RNA-targeted analyses, the absence of DNA contamination was confirmed by RT-PCR without reverse transcriptase. High-throughput sequencing and phylogenetic characterization were performed as described in Section SB.5.

For lipid extraction, frozen sediments were freeze-dried, and 6–20 g samples were extracted ultrasonically 7 times using the Bligh and Dyer method^27^ modified with 0.1 M phosphate buffer (pH 7.4). The organic phase was separated and divided into halves for archaeal and bacterial lipid analyses. Bacterial lipids were analyzed either as degradation products after periodic acid treatment or as intact molecules. Full methods for lipid separation, identification, quantification, and compound-specific δ^13^C analysis are described in Section SB.6. For incubated sediments, the uptake of ^13^C into lipids was assessed by calculating the change in the δ^13^C value before and after incubation, i.e., Δδ^13^C = δ^13^C_final_ – δ^13^C_initial_.

### Estimation of Methane Oxidation Rates Using ^13^C-Labeled Methane

**Figure 1:**
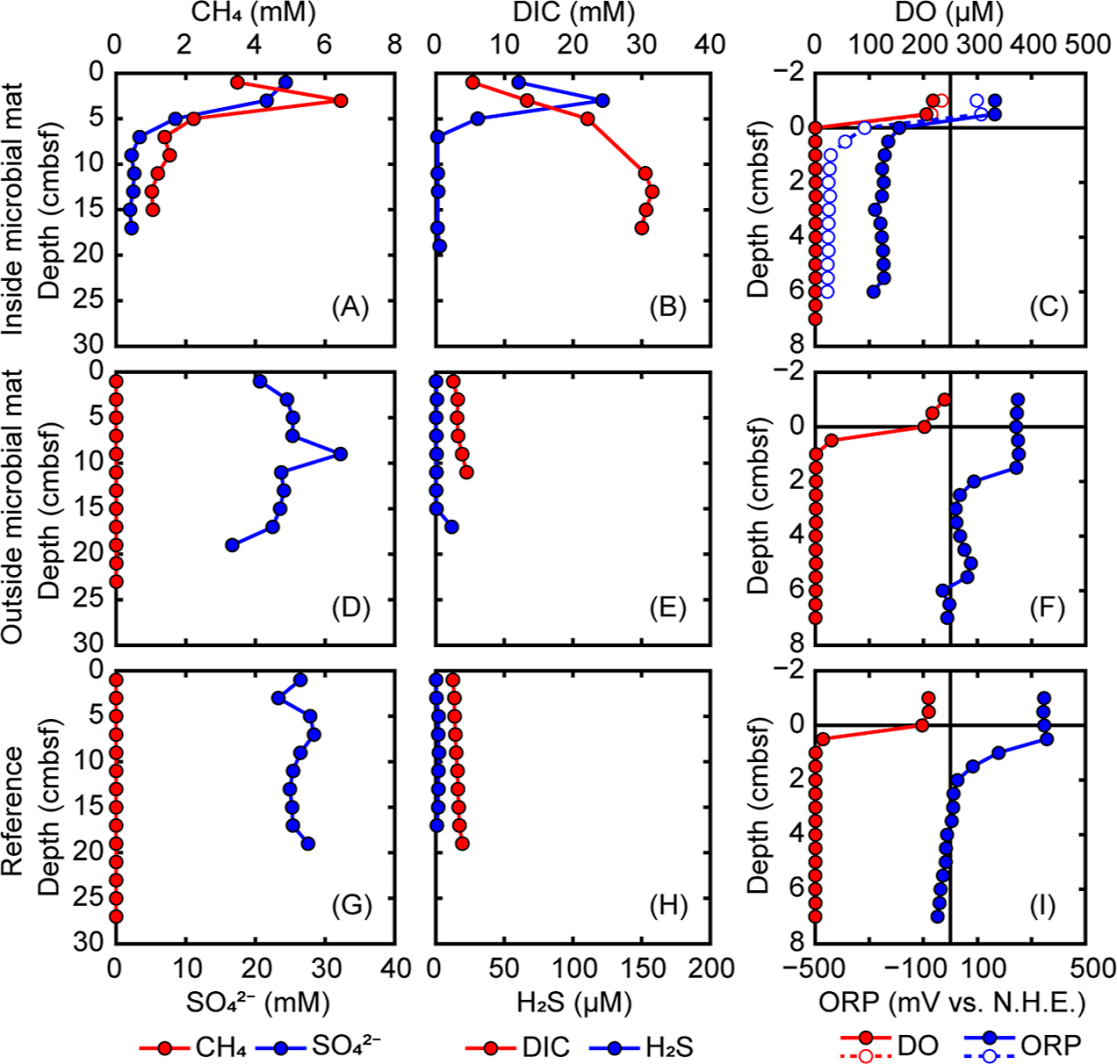
Geochemical profiles of the sediments. (A–I) Concentrations of methane, pore-water sulfate, DIC, hydrogen sulfide, DO, and ORP in sediments of the inside microbial mat (A–C), outside microbial mat (D–F), and reference sites (G–I). Sulfate concentrations are from a previous study.^20^ Note the difference in vertical scale for C, F, and I. Vertical lines in C, F, and I indicate an ORP of 0 mV. N.H.E., normal hydrogen electrode.

For the inside microbial mat sediments, both the oxygen amended and anaerobic incubations were conducted using subsamples of 0–10 cm below the seafloor (bsf), while only anaerobic incubation was conducted using subsamples of 10–14 cmbsf. The subsample division was based on distinct trends of the geochemical parameters (Figure 1). Because such trends were not found in the outside microbial mat and reference sites, the sediments were divided owing to the sediment lithologies; the upper 0–6 cm subsamples were used for oxygen amended incubation and the lower 6–10 or 6–12 cm subsamples were used for anaerobic incubation (Table S2). The potential methane oxidation rate was calculated from the ^13^C-enrichment of DIC in the sediments amended with ^13^C-labeled methane relative to those amended with nonlabeled methane, following the method of Katayama et al.^28^ (the parameters used are listed in Table S2). Uncertainty of the calculated methane oxidation rate was estimated by using the standard error of the slope of the δ^13^C values. A more detailed method for ^13^C-tracer incubation is described in Section SB.7. High-sensitivity rRNA-SIP of ANME archaea was performed on RNA extracted from slurries at the end of the anaerobic incubation (details in Section SB.8).

## Results

### Geochemical Characterization of Sediments

The vertical methane concentration profile in the inside microbial mat sediments showed a maximum (6.5 mM) at 3 cm below the seafloor (bsf) and a decreasing trend down to 13 cmbsf (Figure 1A), while the stable carbon isotopic composition (δ^13^C value) of methane showed an increasing trend around this interval (Figure S3A). The methane concentrations determined in the sediments might be near a minimum due to the degassing during the core recovery. The upward fluid flow rate was determined as the Darcy velocity to be 7.98 ± 1.60 × 10^–8^ m s^–1^ (Section SA.3; Figures S4 and S5). Given the obtained flow rate and average methane concentration (2.0 mM), the methane efflux rate was estimated to be 14 ± 3 mmol^–1^ m^–2^ d^–1^, which is representative of a low flux methane seep.^7^ This low flux regime was supported by the lack of gas seeps on the seafloor as confirmed by the acoustic surveys.^19^ On the other hand, the methane concentrations were near zero (<0.005 mM) in the outside microbial mat and reference site sediments (Figure 1D,G).

The sulfate in pore water decreased from 24 mM at the surface to 2 mM at 9 cmbsf or below in the inside mat sediments (Figure 1A), while the pore-water sulfide concentrations showed a maximum of 121 μM at 3 cmbsf and decreased to <3 μM below 5 cmbsf (Figure 1B). DIC increased with depth from 5 to 30 mM (Figure 1B). The downcore decrease in sulfate, accumulation in sulfide, and increase in the δ^13^C values of methane suggested the occurrence of AOM in the sediments (Section SA.2). Meanwhile, rather constant and/or trace concentrations of sulfate (20–30 mM), sulfide (<2 μM), and DIC (3–5 mM) were detected in the pore water of the outside mat and reference site sediments (Figure 1D,E,G,H).

Depth profiles of the DO concentration and ORP showed a sharp change in redox conditions at the top (0.0 cmbsf) of the inside mat sediments. While the DO concentrations were ∼200 μM in seawater, they were below the detection limit (0.3 μM) in the top and deeper sediments (Figure 1C). Because the microsensor was moved with an interval of 0.5 cm, the actual seawater–sediment interface at the inside microbial mat would be located between 0.0 and −0.5 cm. Using the DO profile and porosity data (Table S2), the minimum diffusive oxygen uptake (DOU)^29^ was estimated to be 1.98 ± 0.02 mmol m^–2^ d^–1^. In the outside mat and reference site sediments, the DO concentrations decreased gradually and were close to the detection limit at >1.5 cmbsf (Figure 1F,I). ORP also changed drastically from positive to negative values (down to −318 mV) compared with the normal hydrogen electrode measurement at the surface of the inside mat sediments, while it decreased gradually to near zero or negative values at 2.0 cmbsf in the outside mat and reference site sediments (Figure 1C,F,I).

### Microbial Community and Lipid Composition in the Sediments

**Figure 2:**
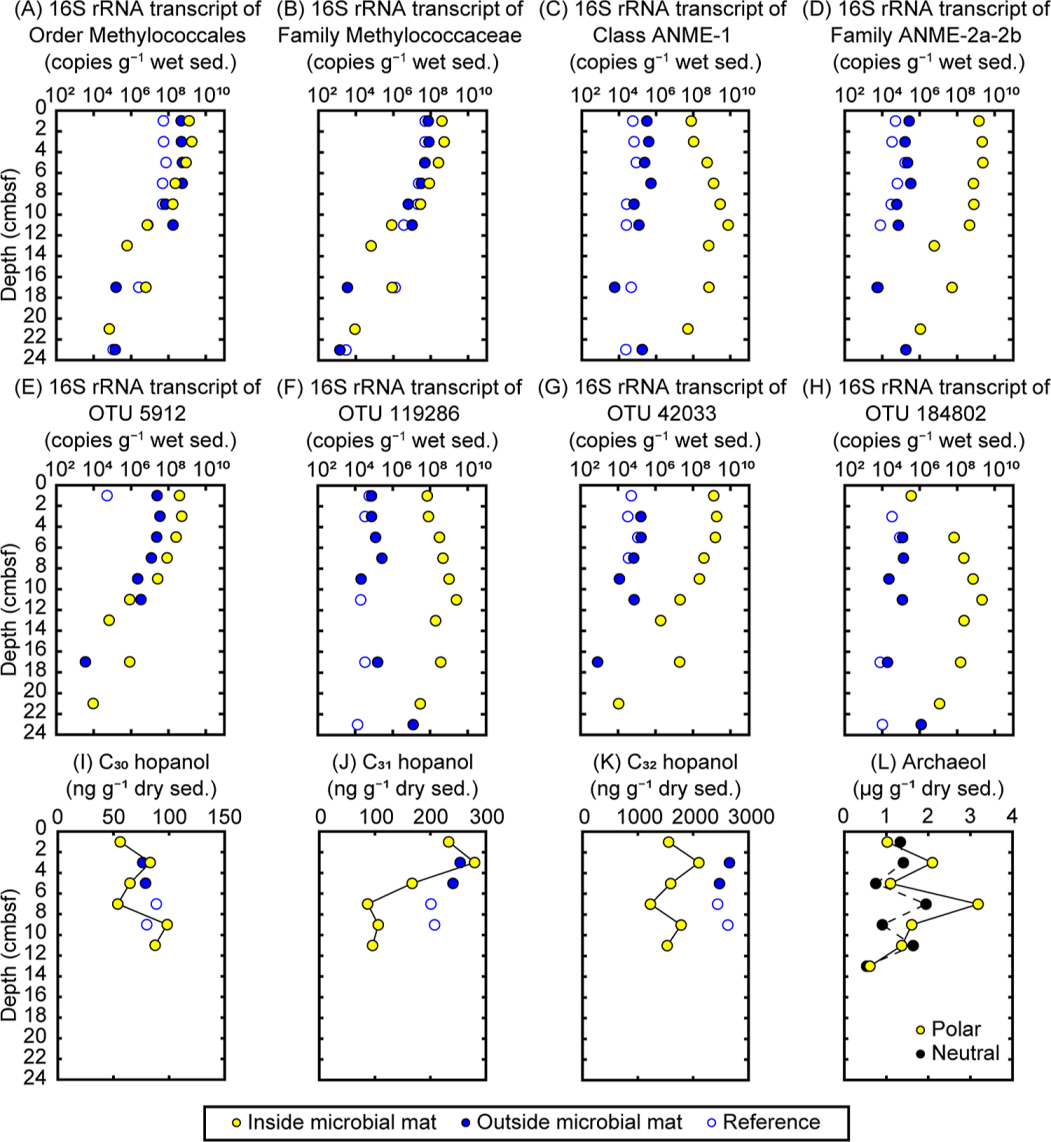
*In situ* vertical distribution of the 16S rRNA transcript copies and lipid biomarkers of aerobic and anaerobic methanotrophs. (A–D) Estimated 16S rRNA transcript copies of the order Methylococcales (A), family Methylococcaceae (B), class ANME-1 (C), and family ANME-2a-2b (D). (E) Copies of the Methylococcaceae OTU 5912 that increased significantly after oxygen amended incubation of the inside microbial mat upper sediments. (F, G) Copies of OTUs 119286 (ANME-1a) and 42033 (ANME-2a-2b) that incorporated ^13^C-labeled methane after anaerobic incubation of the upper sediments. (H) Copies of OTU 184802 (ANME-1a) that incorporated ^13^C-labeled methane after anaerobic incubation of the inside mat lower sediments. (I–K) C_30_ (I), C_31_ (J), and C_32_ (K) hopanols derived from acid treatment of hexa-, penta-, and tetra-functionalized bacteriohopanepolyols, respectively. (L) Neutral and polar archaeol. Archaeols were detected only in the inside mat sediments. Note the logarithmic scale in A–H. “sed.,” sediments.

The estimated copies of 16S rRNA transcripts and genes from Methylococcales or Methylococcaceae (putative aerobic methanotroph) (Figures 2A,B and S7B,D,F,H) and ANME-1 and ANME-2a-2b (putative anaerobic methanotroph) (Figures 2C,D and S8B,D,F,H) were determined based on total copy numbers and relative abundance data (Section SA.4; Figures S6A,B, S7A,C,E,G, and S8A,C,E,G; Table S3). The transcript copies of Methylococcaceae at 1–9 cmbsf were higher in the inside microbial mat sediments (2.8 × 10^7^ to 5.2 × 10^8^ copies g^–1^ wet sediment) than those in the outside mat and reference site sediments (6.3 × 10^6^ to 8.0 × 10^7^ copies g^–1^), while the gene copies were comparable among these three sites (1.4 × 10^7^ to 1.6 × 10^8^ copies g^–1^). Compared to those at the other two sites, the transcript and gene copies of ANME-1 in the inside mat sediments were remarkably high throughout the sampling depth (7.8 × 10^7^ to 7.8 × 10^9^ and 2.7 × 10^8^ to 3.4 × 10^10^ copies g^–1^, respectively), and peaked at 11 cmbsf. ANME-2a-2b also exhibited high transcript (1.1 × 10^6^ to 2.4 × 10^9^ copies g^–1^) and gene copy numbers (2.2 × 10^7^ to 8.5 × 10^8^ copies g^–1^) in the inside mat sediments, which were 1–5 magnitudes greater than those at the other sites.

At all three sites, bacterial lipids (bacteriohopanepolyols, BHPs) were detected as the acid degradation products, C_30_, C_31_, and C_32_ hopanols (Figure S9). C_31_ hopanol accounted for 5–13% of the three total hopanols (87–280 ng g^–1^ dry sediment) and increased at 1–5 cmbsf in the inside mat sediments (Figures 2J and S10A; Table S4). C_32_ hopanol was the most abundant (>80% of the total; 1230–2700 ng g^–1^) without the clear downcore trends (Figures 2K and S10A; Table S4). C_30_ hopanol concentrations were minor (2–5% of the total) and consistent between sites (54–98 ng g^–1^) (Figures 2I and S10A; Table S4). The common archaeal lipids, i.e. neutral and polar archaeols (Figure S9),^30,31^ were detected throughout the depth of the inside mat sediments, whereas they were not detected in sediments of the outside mat and reference sites (Figure 2L). Concentrations of the neutral archaeol were 0.53–2.00 μg g^–1^ (Table S4), while those of polar archaeol were 0.61–3.20 μg g^–1^, peaking at 7 cmbsf (Figure 2L). Neutral and polar hydroxyarchaeols—specific lipids of certain species of methanogens and ANME^32^—were present only in trace amounts.

### Potential Methane Oxidation Rates

In both the oxygen amended and anaerobic incubations of the inside microbial mat sediments supplemented with ^13^C-labeled methane, DIC showed increasing uptake of the methane-derived ^13^C. In the upper sediments (0–10 cmbsf), the δ^13^C values of DIC showed an ∼2800‰ increase at day 105 of oxygen amended incubation and ∼180‰ increase at day 42 of anaerobic incubation (Figure S11A,D). For the lower sediments (10–14 cmbsf), ^13^C uptake was also observed, albeit at much slower rates, showing an ∼24‰ increase at day 176 of anaerobic incubation (Figure S11G). The long-term incubations with time series sampling showed the robustness of the linear relationships in the δ^13^C values of DIC, suggesting that the cross-feeding effect was obviously not significant. Further, the isotopic compositions were similar between the time courses of the duplicate incubations, confirming the reliability of our data sets. Incubation with nonlabeled methane did not show any increase in the δ^13^C value. Based on the ^13^C enrichment in DIC before day 50, the potential methane oxidation rates in the inside mat sediments were 56,000 ± 4000 pmol mL^–1^ d^–1^ (upper sediments, oxygen amended, day 2–48, *r*^2^ = 0.98), 16,000 ± 1000 pmol mL^–1^ d^–1^ (upper sediments, anaerobic, day 1–42, *r*^2^ = 0.98), and 550 ± 90 pmol mL^–1^ d^–1^ (lower sediments, anaerobic, day 7–42, *r*^2^ = 0.94) (Tables 1 and S2). Under oxygen amended condition, oxygen would be preferentially used to oxidize methane based on the thermodynamic theory.^33,34^ The headspace oxygen concentration of 6.8% at the end of the incubation as well as the linear increase in the δ^13^C values of DIC, supported the continued operation of the aerobic oxidation. However, when excess amounts of the electron donor are supplied, the electrons from the oxidation flow into the energetically less favorable electron acceptors.^35^ Thus, anaerobic methane oxidation was not excluded. The simultaneous occurrence of the aerobic and anaerobic methane oxidation would contribute to the potential rate of oxygen amended incubation. Meanwhile, the maximum rate for the anaerobic oxidation was estimated in anaerobic incubation, where oxygen was eliminated. Consequently, the range of the aerobic methane oxidation rates was calculated 41,000 to 56,000 pmol mL^–1^ d^–1^, in which the lower rate was reasonable and conservative, because the minimum rate for the anaerobic oxidation was not clarified. In contrast, the methane oxidation potentials were very low for the outside mat and reference site sediments. The δ^13^C values of DIC did not change significantly until day 34 and 152 in the oxygen amended and anaerobic incubations, respectively (Figure S11B,C,E,F).

**Table 1:** Potential Methane Oxidation Rates and Consumed Methane Proportion in the Inside Microbial Mat Sediments

sediment (cmbsf)	condition	active methanotroph (OTU)	methane oxidation rate (pmol mL^–1^ d^–1^)	methane consumed (% of total flux)^c^
upper (0–10)	oxygen amended	Methylococcaceae (5912), ANME-2a-2b (42033), ANME-1a (119286, 184802)	56,000 ± 4000	
upper (0–10)	anaerobic	ANME-2a-2b (42033), ANME-1a (119286, 184802)	16,000 ± 1000	33.2 ± 2.9 (30.1 ± 2.6) to 33.1 ± 2.8 (28.9 ± 2.5)
upper (0.0–0.5, 0–6)^a^	aerobic	Methylococcaceae (5912)	41,000 ± 4000 to 56,000 ± 4000	0.9 ± 0.1 (10.3 ± 0.9) to 1.3 ± 0.1 (13.7 ± 0.9)
lower (10–22)	anaerobic	ANME-1a (119286, 184802)	550 ± 90^b^	1.4 ± 0.2 (1.3 ± 0.2) to 1.4 ± 0.2 (1.2 ± 0.2)

a Depth distribution of Methylococcaceae was either 0.5 cm given the DO profile, or 6 cm given the distribution of 16S rRNA transcripts and lipids.

b Estimated using 10–14 cmbsf sediments (Table S2).

c Total methane flux was calculated as the sum of the estimated methane efflux (13.8 ± 2.8 mmol m^–2^ d^–1^) and the depth-integrated methane oxidation rates (Table S8). Values in parentheses were calculated using the aerobic oxidation rate integrated for 6 cm.

### Active Methanotrophs Identified by rRNA-SIP and Their *In Situ* Distribution

During the ^13^C-tracer incubation of the inside microbial mat sediments, the numbers of 16S rRNA transcript and gene copies were almost unchanged (Figure S12A,B; Table S5). Overall composition of the transcripts was similar before and after incubation, while the gene composition changed considerably (Figure S12C–E). These results indicated that the transcripts in the tracer experiment were derived from microbial species active *in situ*, whereas the genes may reflect the effect of the incubation setting. Although the transcript copies of Methylococcales did not change significantly, the operational taxonomic unit (OTU) 5912 related to the aerobic methanotroph *Methylobacter psychrophilus*(36) (accession no.: NR_025016, sequence similarity: 93.6%), a species of Methylococcaceae, was metabolically activated during oxygen amended incubation (upper sediments). The estimated number of OTU 5912 transcripts increased from 8.4 × 10^6^ to 4.8 × 10^7^ copies g^–1^ wet sediment (Figure S13D), as determined from the total copies and relative abundances (Figures S12B and S13B). The transcript copies of the other major OTU 193109 (*Methylosphaera hansonii*,^37^ NR_134169, 91.7%) affiliated with Methylococcales did not increase but remained relatively constant during incubation (3.0–8.0 × 10^7^ copies g^–1^). The gene copies of these OTUs exhibited a trend similar to that of the transcript copies (Figures S12A and S13A,C). Although some of aerobic methanotrophs could utilize nitrate/nitrite as the electron acceptors and/or sinks,^38^ the nitrogen pool was apparently low and the phylogenetically relevant taxon, e.g., NC10, was almost not found, strongly suggesting that the nitrate/nitrite dependent methane oxidation was marginal. Methane oxidation related to iron reduction was recently suggested for *Methylobacter* species,^39^ which would warrant future investigation. For anaerobic incubations, high-sensitivity rRNA-SIP demonstrated the incorporation of methane ^13^C into ANME archaea (Section SA.5). Significant enrichment in ^13^C were found in OTUs 119286 (ANME-1a,^32^ AF134392, 97.2%) and 42033 (ANME-2a-2b,^40^ FJ555678, 99.2%) during upper sediment incubation and in OTU 184802 (ANME-1a,^41^ AY714817, 98.0%) during lower sediment incubation (Figure S14 and S15C,D). The effect of the incubation settings, including the long incubation times, was not serious, especially for the transcript assays (Figure S12C–E), highlighting the effective experimental design for the first demonstration of ^13^C probing of ANME, a representative of slow growers.

We determined the *in situ* vertical distribution of the active methanotroph OTUs accordingly (Figures 2E–H, S6B, S7K,L, and S8K,L,O,P,S,T). In the inside mat sediments, the transcript copies of the aerobic methanotroph OTU 5912 at 1–9 cmbsf were more abundant (2.6 × 10^7^ to 5.1 × 10^8^ copies g^–1^) than those in the outside mat and reference site sediments (5.1 × 10^4^ to 3.5 × 10^7^ copies g^–1^) (Figures 2E and S7L). At all three sites, the copy numbers of OTU 5912 decreased with depth, similar to the trend observed for OTU 193109 (Figure S7P). A distinctive distribution of ANME species transcripts was found in the inside mat sediments (Figures 2F–H and S8L,P,T). For ANME-1a, the transcript copies of OTU 119286 were high (3.0 × 10^7^ to 2.5 × 10^9^ copies g^–1^) throughout the sampled depth. The copies of OTU 184802 were rather low (3.5 × 10^5^ copies g^–1^) at 1 cmbsf but increased with depth, peaking at 2.1 × 10^9^ copies g^–1^ at 11 cmbsf. High transcript numbers were detected for the ANME-2a-2b OTU 42033 at 1–9 cmbsf (2.2 × 10^7^ to 1.9 × 10^9^ copies g^–1^), while its transcript levels decreased gradually to a minimum of 1.1 × 10^4^ copies g^–1^ at 21 cmbsf. In the outside mat and reference site sediments, the transcript copies of these ANME OTUs were much less abundant than those in the inside mat sediments, being in the range of 10^2^ to 10^6^ copies g^–1^ (Figure 2F–H).

### Lipid Biomarkers of the Active Methanotrophs

C_30_, C_31_, and C_32_ hopanols were detected in oxygen amended incubation of the upper sediments of the inside microbial mat (Figure S10B). We also detected intact BHPs, consisting of aminopentol, aminotetrol, aminotriol, and bacteriohopanetetrol, supporting the use of these hopanols as proxies of BHP components (Figures S9, S16, and S17). The hopanol concentrations increased over 105 days of incubation, while the relative abundances were almost constant (Table S7). A significant enrichment of ^13^C was observed in C_31_ hopanol after 105 d of labeled incubation, showing Δδ^13^C values of >150‰ (Figure S18A). C_32_ hopanol also showed ^13^C-enrichment, albeit with smaller Δδ^13^C values (>12‰). C_30_ hopanol concentrations were too low to determine its Δδ^13^C values. The nonlabeled incubation did not show any enrichment in ^13^C in the hopanols.

We assessed the *in situ* distribution of these biomarkers and found that the increase in C_31_ hopanol concentrations above 7 cmbsf in the inside mat sediments corresponded to the increase in Methylococcaceae and OTU 5912 transcript copies (Figure 2B,E,J), suggesting that the increase in C_31_ hopanol was caused by methanotrophs producing the precursor aminotetrol. This was distinct from the previous findings that pure cultures of gammaproteobacterial methanotrophs predominantly yielded aminopentol (Figure S10B),^42−44^ suggesting the possibility of C_31_ hopanol as biomarkers of Methylococcaceae bacteria in the sediments. High concentrations of C_32_ hopanol, the precursors for which were aminotriol and bacteriohopanetetrol, were detected throughout the sampling depth, not specifically in the upper parts of the sediments, indicating that it would originate from not only methanotrophs but also other bacteria, such as sulfate reducers.^45^

As for the archaeal lipids in the anaerobically incubated inside mat sediments, the Δδ^13^C values of the archaeols were similar between the labeled and nonlabeled incubations, indicating little uptake of the methane ^13^C into the archaeols (Figure S18B,C). This could be due either to the low levels of lipid synthesis in the ANME cells or to the large contribution of fossil lipids in the sediments, as also argued in previous studies.^46−48^

## Discussion

**Figure 3:**
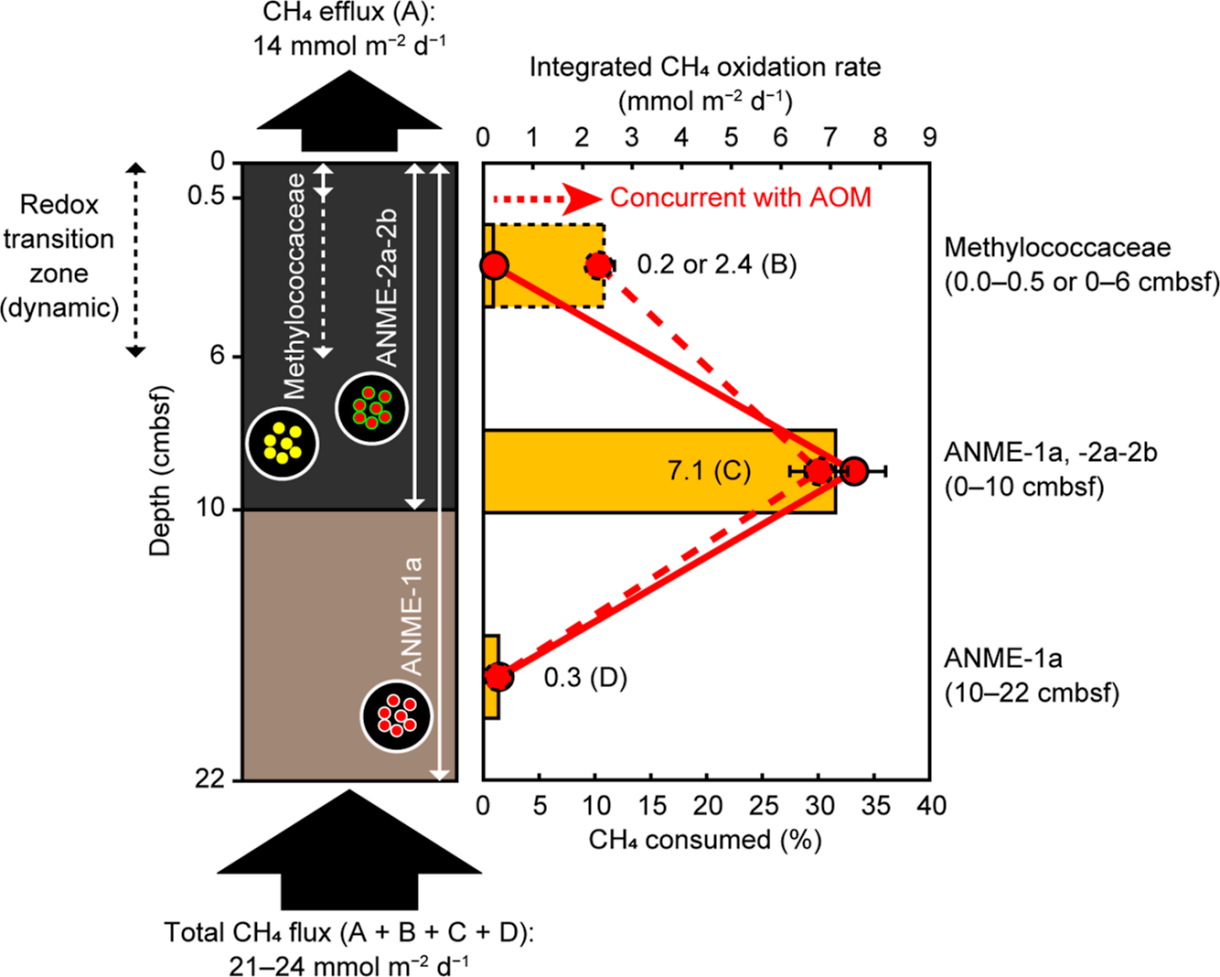
Model of methane consumption processes in the inside microbial mat sediments, showing the impact of concurrent aerobic and anaerobic methanotrophy at the dynamic redox transition zone. Left panel illustrates the *in situ* vertical distribution of key methanotrophs in the upper and lower sediments. Note that the 16S rRNA transcripts of Methylococcaceae were preferentially detected down to 6 cmbsf (dashed arrow, corresponding to the redox transition zone), despite the depletion in DO at <0.5 cmbsf (solid arrow). The total methane flux was estimated by summing the methane exited from the seafloor (efflux) and methane consumed in the sediments (depth-integrated oxidation rates). Right panel shows the depth-integrated methane oxidation rates (bars) and the proportion of methane consumed relative to the total methane flux (circles and lines). For the integrated aerobic methane oxidation rates, two distinct cases are shown: one is inferred from the depth profile of the active aerobic methanotroph species (dashed line) and the other is inferred from the DO profile (solid line). The conservative aerobic oxidation rate (41 nmol mL^–1^ d^–1^) in oxygen amended incubation was used. For the integrated anaerobic oxidation rates, the depth interval was inferred from the depth profile of the ANME-1 and ANME-2 species 16S rRNA transcripts. Error bars were calculated from the errors in the methane oxidation rates.

Despite the depletion of DO (Figure 1C), active aerobic methanotrophs occurred between 0 and 10 cmbsf of the inside microbial mat sediments. The rRNA transcripts of Methylococcaceae OTU 5912 occurred at >10^8^ copies g^–1^ at 1–5 cmbsf, which predominated down to 9 cmbsf compared to those at the other two sites (Figures 2E and S7L). In the same depth interval, we observed remarkable rRNA transcript contents for Epsilonproteobacteria, including aerobic sulfide oxidizers (7.9–55.2% of the total), which coincided with the dominance of Deltaproteobacteria transcripts (24.8–68.9%), including anaerobic sulfate reducers (Figure S6E). The temporal variability of outflow and inflow rates was observed on the seafloor in gas hydrate areas,^49^ which may induce episodic disturbance of the high-porosity sediments of the inside mat (∼0.8 [v/v], Table S2). The provided DO would be rapidly consumed by aerobic microorganisms, thus maintaining low DO and ORP levels while sustaining AOM metabolism even in the surface sediments (Figure 3). The redox conditions of the inside mat sediments would be changing dynamically, allowing the simultaneous metabolic activation of aerobic and anaerobic methanotrophs.

We used the methane oxidation potentials and *in situ* vertical distributions of the active methanotroph species to model the methane consumption processes of the inside microbial mat sediments. The Michaelis–Menten constants (*K*_m_) for AOM were reported ranging between 1.1 and 37 mM, while a recent study by Marlow et al. used the conservative value of 5.65 mM^50^ to normalize the AOM rates at different methane concentrations assessed so far. Although it is preferential that the *K*_m_ value is determined on a site-by-site basis for accurate AOM rate estimation at the specific situation, normalizing by the conservative *K*_m_ is also effective for the direct comparison among the rates measured in earlier studies. Because this *K*_m_ value is higher than the dissolved methane concentration of 0.34 mM in the incubations and that of 2.0 mM in average *in situ*, the AOM rates in this study would not attain a maximum (*V*_max_) and depend on the dissolved methane concentration. Following the Michaelis–Menten equation,^50,51^ the *in situ* AOM rates were calculated to be 71 ± 6 nmol^–1^ mL^–1^ d^–1^ (upper sediments) and 2.5 ± 0.4 nmol^–1^ mL^–1^ d^–1^ (lower sediments) based on the *in situ* methane concentration (Table S8). For aerobic methane oxidation, the reported *K*_m_ values (<100 μM)^52^ were much lower than the methane concentrations in this study. The range of the estimated oxidation rates of 41 ± 4 to 56 ± 4 nmol^–1^ mL^–1^ d^–1^ (upper sediments) could have attained a *V*_max_ and was adopted without calculation.

The calculated methane oxidation rates were integrated for the depth intervals in which active ANME were identified (0–10 cmbsf for ANME-1 and ANME-2a-2b; 10–22 cmbsf for ANME-1) (Figure 2). Here, we assumed that the oxidation rates between 14 and 22 cmbsf were almost the same as those obtained from the 10 to 14 cmbsf sediment incubation, because the rRNA transcripts from ANME-1 remained at high levels (∼10^8^ copies g^–1^). The relative contribution of AOM to methane consumption was quantified by using the depth-integrated oxidation rates and methane efflux data. We found that 33.2 and 1.4% of the total methane flux were consumed by AOM in the upper 10 cm and lower 12 cm sediments, respectively (Table 1 and Figure 3). The higher proportion of methane consumed in the upper sediments was caused by the availability of high concentrations of sulfate—one of electron acceptors for AOM. Although sulfide accumulation^53^ and intermittent oxidative stress^54^ in the surface sediments were crucial inhibitory factors, the active ANME-1 and ANME-2 species (OTUs 119286 and 42033) along with the syntrophic partners were well adapted to the changing conditions. In the lower sediments, the consumed methane proportion was relatively small but not insignificant, considering the large anaerobic area present in the subsurface. Moreover, the ANME-1 species (OTUs 119286 and 184802) could overcome the limited availability of sulfate in deeper environments. Along with the vertical distribution of the ANME transcripts, our findings highlight the niche differentiation of the ANME-1 and ANME-2 species, even the two ANME-1 species, in this gas hydrate area.

As for aerobic methane oxidation, the most conservatively estimated oxidation rate was first integrated for 0.0–0.5 cmbsf, in which the DO concentrations dropped to the detection limit (Figure 1C; Table S8). The depth-integrated oxidation rate (0.20 mmol m^–2^ d^–1^) was lower than that estimated, assuming that the DOU was caused by the aerobic methane oxidation (DOU/2), indicating the operation of aerobic respiration other than methane oxidation. In this case, we estimated that only 0.9% of the total methane flux was consumed by aerobic oxidation. However, the incubation experiments and *in situ* distribution profile indicated high levels of metabolic activity related to the aerobic Methylococcaceae methanotrophs below 0.5 cmbsf of the inside microbial mat sediments (Figures 2B and S7H), suggesting that the contribution of these methanotrophs was likely higher than previously estimated. The aerobic methane oxidation potential (41 nmol mL^–1^ d^–1^) of the upper 10 cm sediments, number of Methylococcaceae OTU 5912 transcripts (>10^8^ copies g^–1^), and high concentrations of C_31_ hopanol at 1–5 cmbsf (167–280 ng g^–1^) (Figures 2E,J and S7L,P) clearly indicated that aerobic methane oxidation was feasible at least down to 6 cmbsf. Therefore, we integrated the obtained oxidation rate to this deeper interval and found that the consumed methane proportion increased dramatically from 0.9 to 10.3%. Consequently, aerobic and anaerobic oxidations could collectively consume up to 41.6% of the total methane flux (Table 1 and Figure 3). The proportion of the methane consumed, i.e., the microbial methane filter efficiency of the sediments, was lower than that expected at methane seeps with low effluxes,^7^ which was possibly due to the direct and careful determination of methane oxidation rates. Because low methane flux areas are often observed in ocean floors,^7^ concurrent aerobic–anaerobic methanotrophy at the redox transition zones is essential for methane consumption in sediments.

Although the Sakata Knoll is a gas hydrate-bearing area, no gas seeps occur on the seafloor,^19^ implying that methane production beneath the seafloor is large enough to form gas hydrates, but not to generate gas seeps. This unique characteristic indicates that microbial methane oxidation is a key issue for characterizing the low methane flux conditions in surface sediments. Using a combination of the *in situ* geochemical and microbiological profiles and the ^13^C-tracer incubation experiment results, we provided an accurate estimate of methane consumption efficiency. The incubation-based method determined not only the AOM rates but also the aerobic oxidation rates instead of the oxygen uptake rates determined by the conventional method. The proportion of these two methane consumption paths was precisely calculated using the kinetics of the methane oxidation rates under *in situ* methane concentrations. Further, high-sensitivity rRNA-SIP clarified the incorporation of ^13^C-labeled methane by the ANME species, highlighting their active involvement in AOM and niche differentiation under dynamic *in situ* conditions. The effective area of aerobic methane oxidation was carefully revised based on the rRNA and lipid biosyntheses, revealing the substantial contribution of active Methylococcaceae species to methane consumption. Our findings emphasize the importance of concurrent aerobic and anaerobic methanotrophy at the redox boundary in determining the methane flux in marine gas hydrate-bearing sediments.

## Data Availability

All data are available in the main text or the Supporting Information. All the raw 16S rRNA gene and transcript sequences generated in this study were deposited at the DNA Data Bank of Japan (DDBJ) under BioProject PRJDB14216. The 16S rRNA libraries data (246 libraries) can be found under BioSample SAMD00521061–SAMD00521314 (*in situ* experiment DNA, 75; *in situ* experiment RNA, 75; incubation experiment DNA, 30; incubation experiment RNA, 30; SIP experiment RNA, 36).

## Supporting Information Available

Experiment scheme, geological setting of study area, and supplementary results and methods for gas and pore water geochemistry, fluid flow rate, microbial community and lipid composition in the sediments, ^13^C-tracer incubation, and high-sensitivity rRNA-SIP of ANME species (PDF)
List of push core samples, parameters, and methane oxidation rates measured and calculated for incubated sediments, summary of high-throughput 16S rRNA gene and transcript sequence data, concentrations of archaeal and bacterial lipids *in situ* and in cubated sediments, buoyant density and sequence number of density-resolved fractions in the high-sensitivity RNA-SIP, and water contents of sediments (XLSX)
