# Supplementary material for: Impact of Concurrent aerobic–anaerobic Methanotrophy on Methane Emission from Marine Sediments in Gas Hydrate Area
Source: Environ Sci Technol. 2024 Mar 6;58(11):4979–88. doi: 10.1021/acs.est.3c09484 (PMC10956523; doi:10.1021/acs.est.3c09484)
Supplement: Supplementary file 1 — es3c09484_si_001.pdf [file es3c09484_si_001.pdf]

Supporting Information for:

Impact of concurrent aerobic–anaerobic methanotrophy on methane emission from marine sediments in gas hydrate area

Yusuke Miyajima[1]‡, Tomo Aoyagi[2]‡, Hideyoshi Yoshioka[1]*, Tomoyuki Hori[2]*, Hiroshi A. Takahashi[3], Minako Tanaka[4], Ayumi Tsukasaki[2], Shusaku Goto[1], Masahiro Suzumura[2]

[1] Research Institute for Geo-Resources and Environment, Geological Survey of Japan, National Institute of Advanced Industrial Science and Technology (AIST), Central 7, 1-1-1 Higashi, Tsukuba, Ibaraki 305-8567, Japan

[2] Environmental Management Research Institute, National Institute of Advanced Industrial Science and Technology (AIST), 16-1 Onogawa, Tsukuba, Ibaraki 305-8569, Japan

[3] Research Institute of Earthquake and Volcano Geology, Geological Survey of Japan, National Institute of Advanced Industrial Science and Technology (AIST), Central 7, 1-1-1 Higashi, Tsukuba, Ibaraki 305-8567, Japan

[4] KANSO TECHNOS Co., Ltd., 14 Kanda Higashimatsushita-cho, Chiyoda-ku, Tokyo 101-0042, Japan

‡These authors contributed equally to this work: Yusuke Miyajima; Tomo Aoyagi

*Correspondence to: Hideyoshi Yoshioka (hi-yoshioka@aist.go.jp); Tomoyuki Hori (hori-tomo@aist.go.jp)

This file includes:

Pages: 33

Figures: 18

Tables (separate file): 9

SA Supplementary Text

SA.1 Geological setting of study area

The eastern margin of the Japan Sea is characterized by oil and gas accumulation mainly sourced from organic-rich siliciclastic sediments of the Miocene to Pliocene age, which were deposited on igneous rock associated with submarine volcanism during the back-arc opening of the Japan Sea. Tectonic inversion from rifting to compression during the Pliocene resulted in the formation of a number of reverse faults and anticlinal structures, which today comprise the main loci of oil and gas accumulation and shallow gas hydrate occurrence [1-4]. The shallow gas hydrates in the Japan Sea are concentrated within columnar acoustic blanking zones, called gas chimney structures, with diameters of a few hundred meters to a kilometer and thickness of ~100 m below the seafloor [5].

SA.2 Carbon and hydrogen isotopic composition of methane and dissolved inorganic carbon (DIC)

In the inside microbial mat sediments, methane was produced through hydrogenotrophic methanogenesis [6], as evidenced by its carbon and hydrogen isotopic composition. The δ^{13}C and δ^{2}H values were −74.6‰ to −71.9‰ and −164‰ to −158‰, respectively (Figure S3). The δ^{13}C values increased with depth between 1 and 9 cm below the seafloor (bsf), and then decreased with the downcore (Figure S3). A slight increase in the δ^{13}C values could result from the selective uptake of ^{12}C in the methane carbon pool during anaerobic methane oxidation (AOM) (Figure S3). The δ^{13}C values of DIC in the inside mat sediments were −36.7‰ at 3 cmbsf and −20.6‰ at 17 cmbsf, while those in the reference site sediments were generally higher than −10‰. DIC in the outside mat sediments also exhibited a significant depletion in ^{13}C (δ^{13}C values as low as −30‰) (Figure S3).

SA.3 Fluid flow rate

Temperatures of the bottom-water and sediment at the inside microbial mat were measured for seven months (Figure S4). Assuming vertical fluid flow (Darcy flow) with a constant velocity in the sediment, we estimated the thermal diffusivity of the sediment and constant velocity to be $2.69 \pm 0.02 \times 10^{-7}$ m^2 s^{-1} and $7.98 \pm 1.60 \times 10^{-8}$ m s^{-1}, respectively, based on the paired temperature data. We calculated the sediment temperature with the estimated values and measured bottom-water temperature. The calculated time series of the sediment temperature showed good agreement with the measured sediment temperatures (Figure S5).

SA.4 Microbial community composition in the sediments

Copy numbers of 16S rRNA transcripts at 1–11 cmbsf in the inside microbial mat sediments were 4.0×10^{10}–1.3×10^{11} copies g^{-1} wet sediment, almost an order of magnitude higher than those in the outside mat and reference site sediments (2.3×10^{9}–2.6×10^{10} copies g^{-1}), while the gene copy numbers did not vary between the three sites (3.1×10^{9}–5.9×10^{10} copies g^{-1}) (Figure S6A, B). Both gene and transcript copy numbers decreased below 11 cmbsf at the three sites. Principle coordinate analysis (PCoA) of the high-throughput sequence data distinguished microbial communities of the inside mat sediments from those of the outside mat and reference site sediments, suggesting the metabolic activation of the specific microbial constituents at 11–21 cmbsf in the inside

mat sediments (Figure S6C). The transcripts in the inside mat sediments originated mainly from Epsilonproteobacteria (relative abundance: 7.9%–55.2%), Deltaproteobacteria (24.8%–68.8%), Bacteroidetes (2.4%–8.3%), and ANME-1 (0.06%–7.8%) at 1–9 cmbsf, whereas Deltaproteobacteria (52.2%–70.8%) and ANME-1 (8.7%–25.4%) were predominant at greater depths (Figure S6E). Overall composition of the genes was similar to that of the transcripts, though relative abundance varied (Figure S6D). At the outside mat and reference sites, the transcript and gene compositions were almost unchanged with depth of the sediments (Figure S6D, E).

SA.5 High-sensitivity identification of the $^{13}CH_4$-incorporating ANME species
Relative abundances of the microbial community constituents in the first- (‘1H’) and second-heaviest (‘2H’) RNA fractions were compared between the $^{13}CH_4$-labeled and non-labeled anaerobic incubations of the inside microbial mat sediments (Figure S15A−E and Table S6). While there were no obvious differences in the phylum and class level compositions (Figure S15A), the operational taxonomic unit (OTU) level analysis identified 28 OTUs (upper and lower sediments: 18 and 10, respectively) exhibiting >1.2-fold greater abundances in the ^{13}C treatments than those in the non-labeled treatments ($P < 0.05$), which were identified as ^{13}C-incorporating microorganisms (Figure S15B−E). The abundances of OTUs 42033, 119286 (upper sediments), and 184802 (lower sediments) (closely related to the known ANME-2a-2b [accession no.: FJ555678, sequence similarity: 99.2%] and ANME-1a [AF134392, 97.2% and AY714817, 98.0%]) were 0.198%, 1.301%, and 4.655% in the labeled treatments, which were 1.7-, 2.0-, and 1.3-fold higher than those in the non-labeled treatments, respectively (Figure S14 and S14C, D). Furthermore, some of the identified 25 bacterial OTUs were related to sulfate reducers, possibly including the syntrophic partner of the ANME species, whereas others belonged to the heterotrophic and/or fermentative bacteria possibly supported by the ANME-driven microbial food web.

SB Supplementary Methods

SB.1 Gas and pore water analysis
For headspace methane concentration measurements, 27-mL sediments were subsampled immediately after collection into 50-mL glass vials using cut-off syringes, fixed in a saturated NaCl solution with 10 wt% benzalkonium chloride, and sealed with butyl rubber septa and aluminum caps. The vials were stored at room temperature until being subjected to gas chromatography (GC) equipped with a PoraBOND Q column (Agilent, Santa Clara, CA, USA) and a flame ionization detector (FID; GC6890, Agilent). Sulfate concentrations in pore waters were measured by ion chromatography as previously reported [7]. Sulfide concentrations were measured in subsamples treated with 2-N zinc acetate solution using a spectrophotometer (UV-1850, Shimadzu, Kyoto, Japan). Concentrations and $\delta^{13}C$ values of DIC were measured using a Delta V Advantage isotope ratio mass spectrometer (IRMS) equipped with a gas chromatography-based Gas Bench II system (Thermo Fisher Scientific, Waltham, MA, USA). Repeatability of triplicate measurements was generally better than 3% in relative standard deviation for the concentrations and 0.2‰ in standard deviation for the $\delta^{13}C$ values. The stable carbon and hydrogen isotopic compositions ($\delta^{13}C$ and $\delta^{2}H$ values vs. VPDB and VSMOW, respectively) of methane were measured by gas chromatography combustion isotope ratio

mass spectrometry (GC-C-IRMS, Trace GC-GC IsoLink-Conflo-Delta V Plus, Thermo Fisher Scientific). Measurements were repeated in triplicate, with standard deviation of <0.4‰ and <1.6‰ for $\delta^{13}C$ and $\delta^{2}H$, respectively. For the geochemical and microbiological depth profiles, each data point was plotted at the center of the 2-cm core interval.

SB.2 Microsensor analysis of sediments

Depth profiles of the DO concentration and ORP were obtained immediately after sample collection using a profiling system (x-5 UniAmp, Unisense A/S, Aarhus, Denmark) with Micromanipulators MM33-2 (Unisense A/S) equipped with an oxygen microsensor and a redox and reference microelectrodes, all of which have a tip diameter of 100 μm. Since the upper part of the push core sample was filled with *in situ* seawater, DO and ORP were measured from the overlying seawater at 10 mm above the top of the sediments to a depth of 70 mm below the top. The microsensors were moved at a 5 mm interval with a speed of 10 mm s^{-1} after measurement at each depth.

SB.3 Measurement of the sediment and bottom-water temperatures

A stand-alone heat flow meter (Kaiyo Denshi Co., Ltd., Saitama, Japan) was installed at the microbial mat site in the Sakata Knoll for 7 months. One of thermistors was set at 7.4 cmbsf to monitor sediment temperature at a resolution of 0.001 K. A water temperature recorder RBR solo^{3}T (RBR, Ltd., Ottawa, ON, Canada) was deployed on the seafloor near the microbial mat, recording at resolutions lower than 0.0005 K.

SB.4 Estimation of pore fluid flow rate

Fluctuations in bottom-water temperature are propagated downward, causing fluctuations of the sediment temperature beneath the seafloor [8-10]. Assuming pore fluid flow (Darcy flow) [11] of a constant rate v_f in homogeneous sediments saturated with fluid, sediment temperature T at depth z is expressed by the Fourier series of time t given as [11]:

$$T(z,t) = \sum_i A_i \exp\left(\frac{vz}{2\kappa_m} - \frac{z}{2\kappa_m}\sqrt{\frac{\alpha_i + v^2}{2}}\right) cos\left(\frac{2\pi t}{P_i} - \theta_i - \frac{z}{2\kappa_m}\sqrt{\frac{\alpha_i - v^2}{2}}\right) \quad (1)$$

where A_i and θ_i are the amplitude and phase of the temperature fluctuation of period P_i, respectively. Parameters v and α_i in equation (1) are defined as:

$$v = \frac{(\rho c_p)_f}{(\rho c_p)_m} v_f \quad (2)$$

$$\alpha_i = \sqrt{v^4\left[1 + \left(\frac{8\pi\kappa_m}{P_i v^2}\right)^2\right]} \quad (3)$$

where κ_m is the thermal diffusivity of sediments, and $(\rho c_p)_f$ and $(\rho c_p)_m$ are the heat capacity of pore fluid and sediments, respectively.

For estimating κ_m and v_f from the long-term bottom-water and sediment temperature data, we first calculated A_i and θ_i for period P_i from the bottom-water temperature fluctuation data using the discrete Fourier transform. Then, κ_m and v were estimated from the sediment temperature fluctuation data using equations (1) to (3) with the Bayesian inversion method [12]. Finally, v_f was calculated from the inverted value of v using equation

(2). In the calculation, we assumed $(\rho c_p)_f$ to be 4.093 MJ m^{-3} K^{-1}, that is, the heat capacity of seawater [13]. We calculated $(\rho c_p)_m$ from the inverted value of κ_m using the empirical relationships of marine sediment thermal properties [14].

SB.5 High-throughput sequencing and data processing

The sediment samples were centrifuged for 5 min at 15,300 *g* and 4 °C, then the wet weight of the resulting pellets was measured. The dry weight for the representative sediment samples was determined by freeze-drying and used to calculate the water content (Table S9). The PCR and RT-PCR amplicons were purified first with an AMPure XP Kit (Beckman Coulter, Tokyo, Japan) and afterwards with a Wizard SV gel and PCR clean-up kit (Promega). The DNA concentration of the purified amplicon was determined spectrophotometrically with a Quant-iT PicoGreen dsDNA reagent and kit and a NanoDrop 3300 fluorospectrometer (Thermo Fisher Scientific). An appropriate amount of the purified amplicon (i.e., barcode-encoded library) and an internal control (PhiX Control V3; Illumina, San Diego, CA, USA) were subjected to paired-end sequencing with a 300-cycle MiSeq Reagent kit and MiSeq sequencer (Illumina). The PhiX, low-quality ($Q < 30$) and chimeric sequences were removed, and paired-end sequences were assembled, as previously described [15, 16]. The sequences in each sequence library were phylogenetically characterized using QIIME software version 1.7.0 [17] with the Greengenes 16S rRNA database version 13_8. We constructed 150 libraries that consisted of 54 libraries at the inside microbial mat sediments (9 depths × 3 replications × 2 nucleic acid types [gene and transcript]), 48 libraries at the outside mat sediments (8 depths × 3 replications × 2 nucleic acid types), and 48 libraries at the reference site sediments (8 depths × 3 replications × 2 nucleic acid types) (Table S3). In addition, 60 libraries from the incubation samples were comprised of 12 libraries from the start of the incubation (2 conditions [upper and lower] × 3 replications × 2 nucleic acid types) and 48 libraries from the ends of the incubation (3 conditions [upper oxygen amended, upper anaerobic, and lower anaerobic] × 2 replications × 2 technical replications × 2 treatments [^{13}C-labeled and non-labeled] × 2 nucleic acid types) (Table S5). The sediment and incubation sample libraries included 13.9 million (average of 25,032 per library) and 5.7 million (average of 94,756 per library) sequences, respectively. Using QIIME software, α-diversity indices (i.e., Chao1, Shannon, and Simpson reciprocal) and the weighted UniFrac distances for principal coordinate analysis were calculated based on equal numbers of sequences (n = 41,514 and 31,349 for the sediment and incubation slurry samples, respectively). OTUs were defined using a cut-off of 97% sequence identity.

SB.6 Lipid separation, identification, quantification, and carbon isotope analysis

The archaeal halves were chromatographically separated on a silica gel column into neutral core lipids (*n*-hexane/ethyl acetate fraction) and intact polar lipids (IPLs, methanol fraction) by modifying the method of Oba *et al*. [18]. The IPLs were hydrolyzed with HCl. Bacteriohopanepolyols (BHPs) were separated from the bacterial halves through tightly-packed glass wool with chloroform/methanol and degraded into GC-amenable terminal hopanols with periodic acid (H_5IO_6) and sodium borohydride ($NaBH_4$) as previously described [19, 20]. The archaeols and BHP-derived hopanols were silylated with *N*,*O*-bis(trimethylsilyl)trifluoroacetamide (BSTFA) and analyzed using gas chromatograph-mass spectrometers (GC–MS; GC6890N-5973 Network MSD and

GC7890B-5977B MSD, Agilent). The relative abundance of C_{30}, C_{31}, and C_{32} hopanols was determined based on the peak areas on the extracted ion chromatograms for *m*/*z* 191. Compounds were quantified using GC7890 and GC8890 systems (Agilent) equipped with a FID, with a standard deviation better than 4%. The concentrations of C_{30} and C_{31} hopanols were calculated from their relative abundance to C_{32} hopanol determined by GC–MS. Stable carbon isotopic composition of the silylated lipids in the incubated sediments was measured using a GC-C-IRMS (GC5890 [Agilent] coupled to a Delta Plus Advantage [Thermo Fisher Scientific] with a combustion interface). The isotopic composition (in the $\delta^{13}C$ notation vs. VPDB) was calibrated using a mixed *n*-alkane standard of known isotopic composition, with a standard deviation better than 0.9‰. The $\delta^{13}C$ values were not corrected for carbon added by trimethylsilylation. For the oxygen amended sediments of the inside microbial mat, the BHP fractions of the total extracts were acetylated with acetic anhydride/pyridine and subjected to high performance liquid chromatography mass spectrometry (1100 Series HPLC system coupled to a 6490 Triple Quad MS, Agilent, Santa Clara, CA, USA) equipped with an atmospheric pressure chemical ionization (APCI) source. Compounds were separated using an XDB-C18 column (250 mm × 4.6 mm, 5 μm) maintained at 30 °C. BHPs were identified in positive ion mode using product ion monitoring (PIM) of the protonated molecular ion precursor $[M + H]^+$ with a fragmentor voltage of 380 V and collision energy of 10, 20, and 30 V. Compound identification was based on comparison with published mass spectra [21-23].

SB.7 ^{13}C-tracer incubation

The sediments stored at 4 °C were mixed with the sterile artificial sulfate-, nitrate-, and nitrite-free seawater at a ratio of 1:2 (v/v) [24] and treated with $NaHCO_3$ in an anaerobic chamber. We collected 1 mL of the mixed slurry samples in triplicate for nucleic acid analysis. The mixed slurry was divided into a set of four glass vials in each incubation and strictly sealed with butyl rubber septa and aluminum caps to prevent the leakage of headspace gas. Forty mL (for oxygen amended incubations) and 60 mL (for anaerobic incubations) of the mixed slurry in 120-mL vials were employed. Even if the different volumes were used, the area bordering the gas and liquid phases, as well as the concentrations of gaseous components, were set equally for all the three sites. The headspace of the vials was flushed with oxygen-free nitrogen gas, while 20 vol% each of oxygen (only for oxygen amended incubation) and methane were injected. The methane partial pressure of 20% was equivalent to the dissolved concentration of 0.34 mM at 4 °C in seawater with standard ion concentrations [25]. Because the methane concentrations were different among the three site sediments, the comparable concentration for all the incubation experiments was employed. Non-labeled methane with a $\delta^{13}C$ value of −42.99‰ was injected into two vials (M1 and M2), while ^{13}C-labeled methane (99% ^{13}C) and non-labeled methane (1:4 v/v) were injected into the remaining two vials (13M1 and 13M2). Although not ideal, the duplicate incubations were the best strategy possible to meet the needs for geochemical and microbiological assays. For anaerobic incubation, titanium citrate was added as a reducing agent. The remaining slurry was used for biomarker analysis of the initial sediments. All the vials were incubated under static conditions in the dark at 4 °C. Incubation was stopped by opening the vials. After duplicate sampling for nucleic acid analysis, the sediment slurry was transferred for biomarker analysis of the final sediments. The time series sampling was performed non-

destructively using a syringe with particular care to prevent the gas and liquid phase contamination. Changes in headspace methane, nitrogen, and oxygen concentrations in the vials were monitored with a GC-8A (Shimadzu) equipped with a thermal conductivity detector (TCD). The oxygen amended incubation was stopped when methane and oxygen concentrations decreased to ~13 and 6.8 vol%, respectively. The aqueous phase was collected from the vials to monitor changes in the carbon isotopic composition of DIC measured as described in Section SB.1.

SB.8 High-sensitivity rRNA-SIP of ANME archaea
One set of the total RNA extracted in duplicate from the anaerobic incubation slurries with ^{13}C-labeled and non-labeled methane at day 147 (upper sediments) and day 176 (lower sediments) was selected according to high similarities in community composition (Figure S12C). The total RNA concentration was determined in triplicate using a RiboGreen RNA quantification kit (Thermo Fisher Scientific) and microplate reader (SH-900Lab; Corona Electric Co., Ltd., Hitachinaka, Japan). We added 500 ng of RNA to the cesium trifluoroacetate (CsTFA) solution (Wako Pure Chemical Industries, Osaka, Japan) and separated by ultracentrifugation at 128 000 *g* and at 20 °C for >60 h [26]. Density gradients were fractionated, and the CsTFA buoyant density (BD) of each fraction was determined with a refractometer (AR200; Thermo Fisher Scientific) [27]. The first-heaviest ("1H"), second-heaviest ("2H"), and light ('L') fractions of RNA with BDs of 1.798–1.803, 1.791–1.796, and 1.772–1.774 g mL^{-1}, respectively, were subjected to RT-PCR and high-throughput sequencing as described above, except for the 28 cycles employed in the thermal condition of RT-PCR. The total number of 16S rRNA transcript sequences obtained from the 36 density fractions was 4.9 million, corresponding to an average of 136,244 sequences per library (Table S6). Relative OTU abundance was determined in both the labeled and non-labeled incubations, while the statistical significance of their difference was assessed with a Student's *t*-test. The OTUs exhibiting >1.2-fold higher abundances in the heavy fractions of the labeled incubation than those of the non-labeled incubation were phylogenetically identified with the BLAST program in the NCBI nucleotide sequence database (https://blast.ncbi.nlm.nih.gov/blast.cgi). The taxonomy of the archaea OTU assigned by the BLAST program was reanalyzed against the Silva v123 databases [28] with SINA software version 1.2.11 [29].

Supplementary Figures

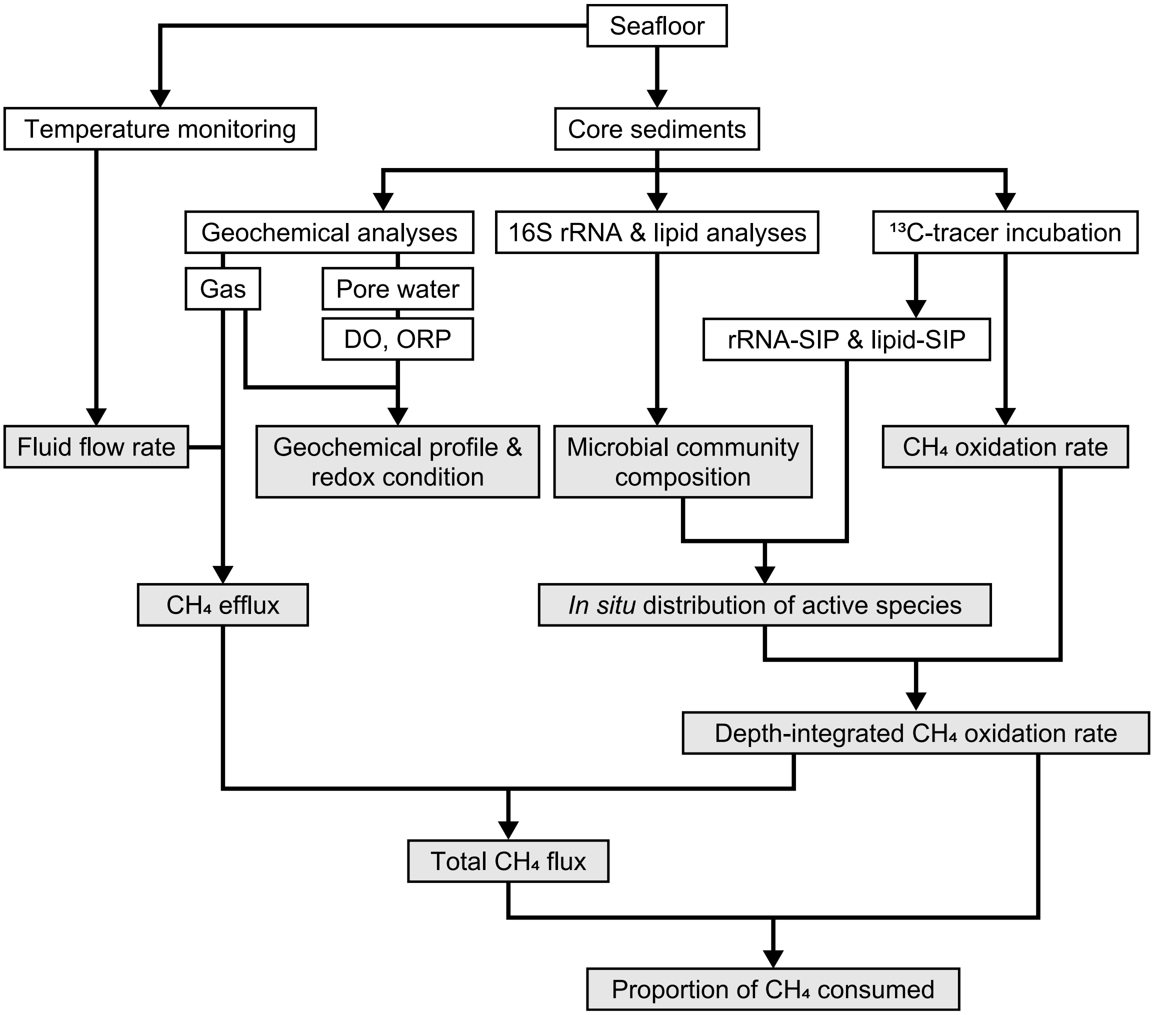

Figure S1 Experiment scheme to investigate the methane consumption process in the marine sediments.

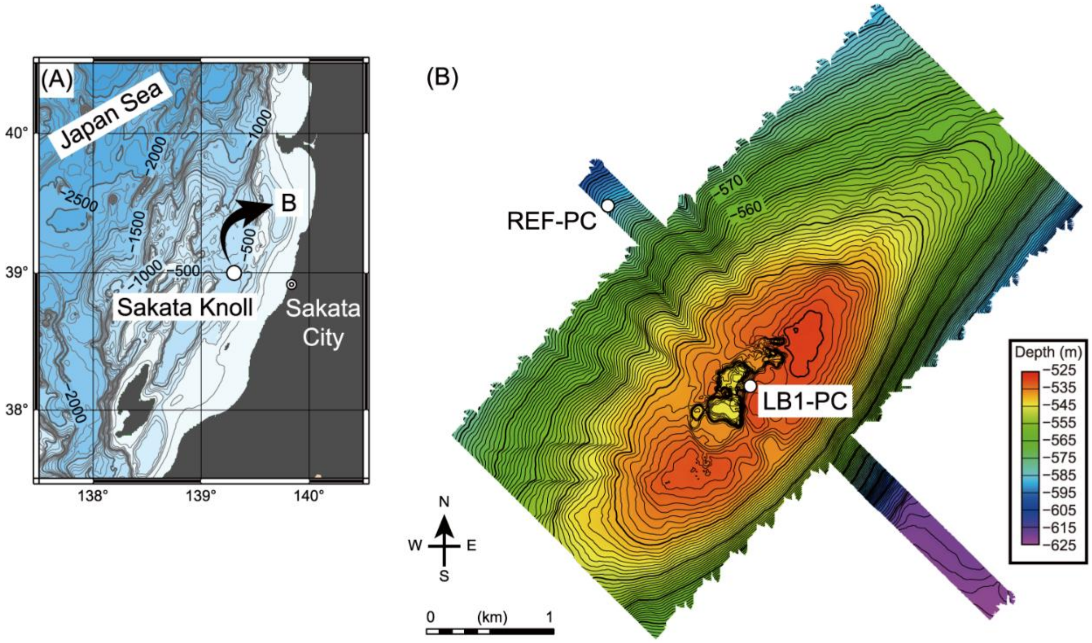

Figure S2 Location of the study sites. (A) Location of the Sakata Knoll (circle). (B) Bathymetric map of the knoll (modified from previous studies [7, 30]). Push coring sites are shown as circles.

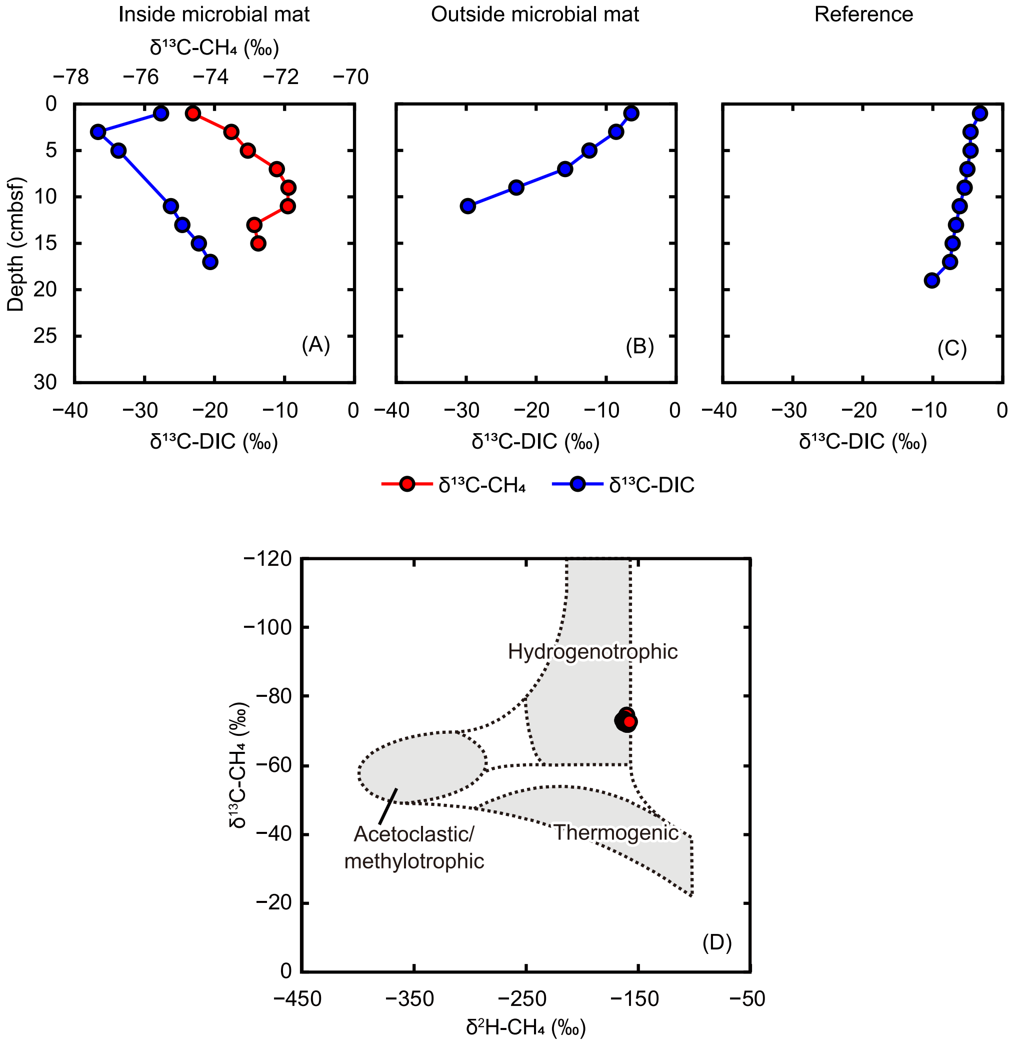

Figure S3 Stable isotopic compositions of methane and dissolved inorganic carbon (DIC) in the sediments. (A–C) Vertical distribution of the carbon isotopic composition of methane and DIC in sediments of the inside microbial mat (A), outside microbial mat (B), and reference site (C). (D) Carbon and hydrogen isotopic compositions of methane in the inside microbial mat (circles). Classification of the methane source is based on Whiticar [6].

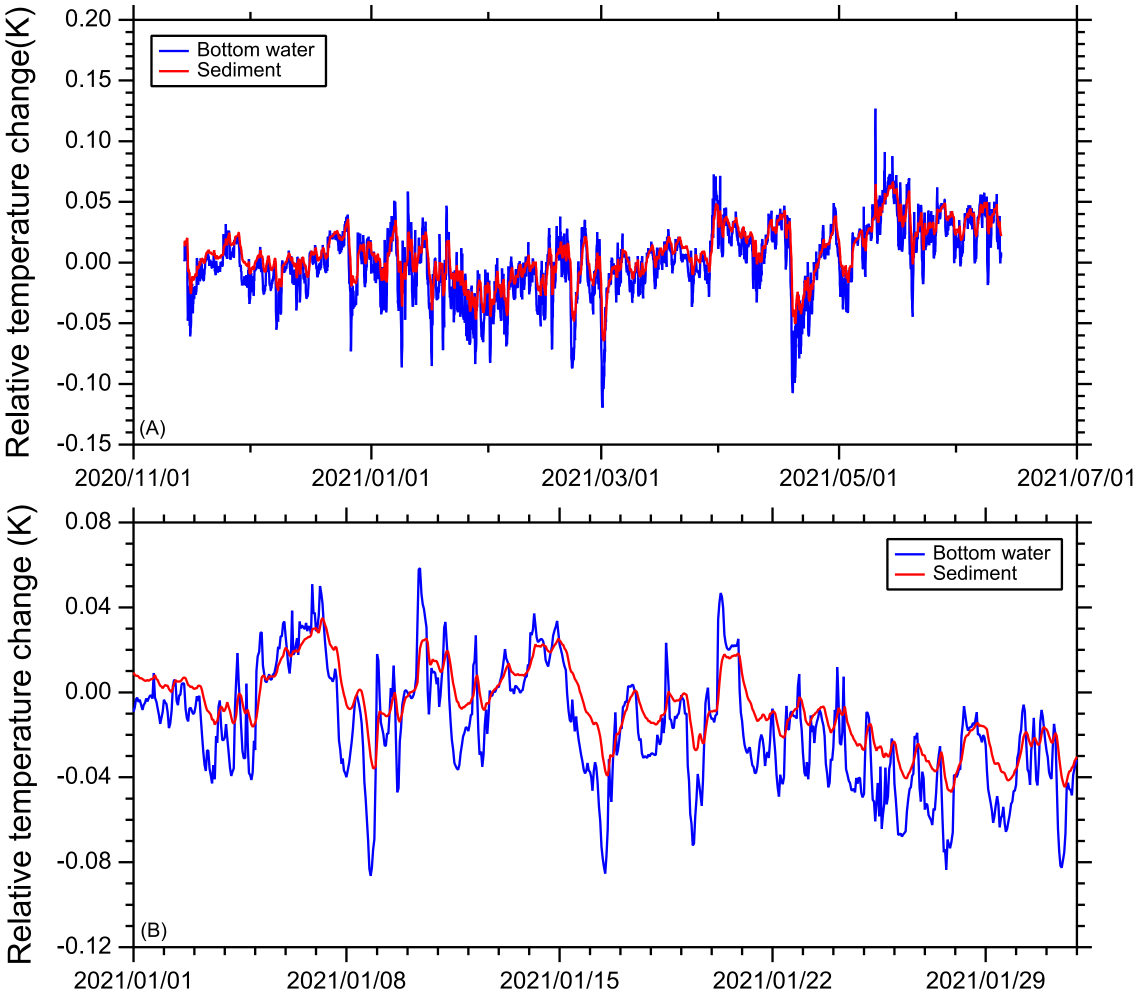

Figure S4 Time series of the bottom-water temperature (blue line) and sediment temperature (red line) at 7.4 cmbsf in the inside microbial mat from November 14, 2020, to June 11, 2021 (A) and during January, 2021 (B). Temperatures are expressed relative to the measurements just before instrument deployment. Vertical scale in B was magnified.

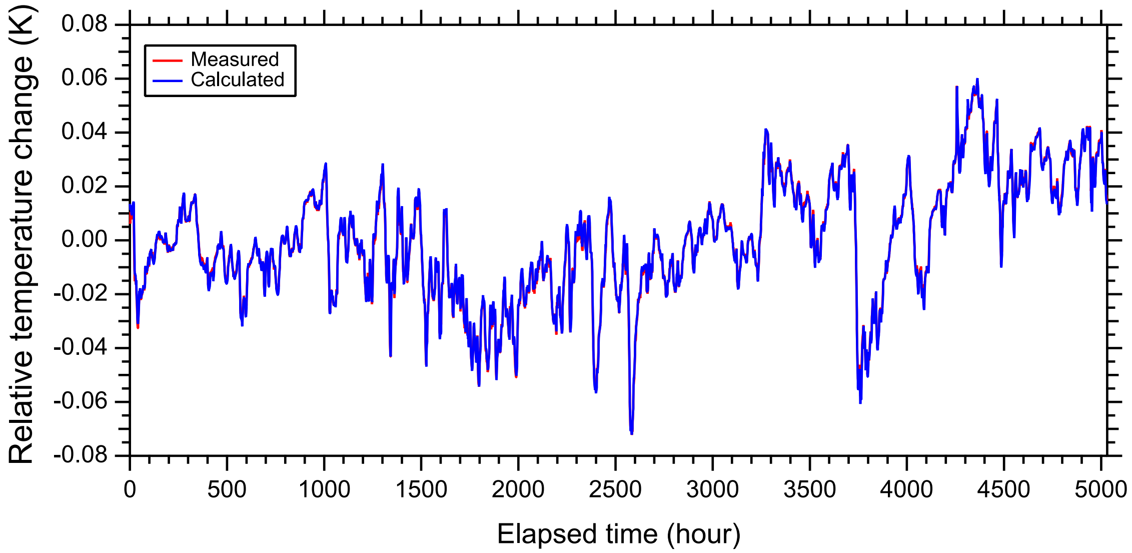

Figure S5 Comparison between the measured (red line) and calculated (blue line) sediment temperatures. The best estimates of κ_m and v_f and the measured bottom-water temperatures were used for the calculation.

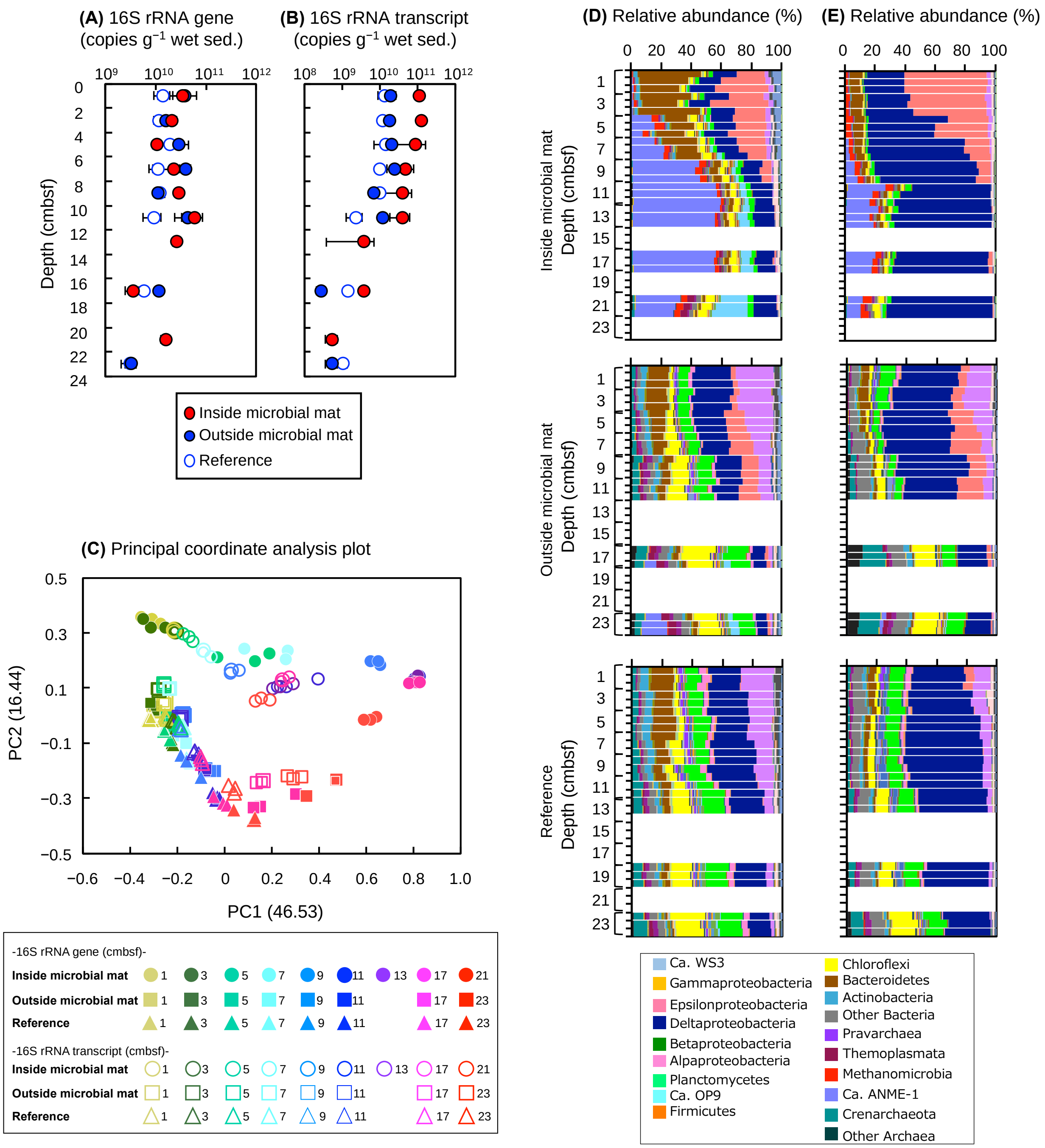

Figure S6 Vertical distribution of 16S rRNA gene- and transcript-based microbial community compositions in the sediments. (A, B) Copy numbers assessed by quantitative PCR (A) and RT-PCR (B) (n = 3). (C) Principle coordinate analysis (PCoA) plot of high-throughput sequence data (n = 3) based on the equal numbers (n = 41,514) of sequences. The sampling depths are indicated by numbers and different colors in the explanatory note. (D, E) Phylogenetic assignment at the phylum and class levels (in different colors) for 16S rRNA gene (D) and transcript sequences (E) generated using the QIIME software version 1.7.051 with the Greengenes 16S rRNA database version 13_8. Detailed information of the determined sequence is shown in Table S3.

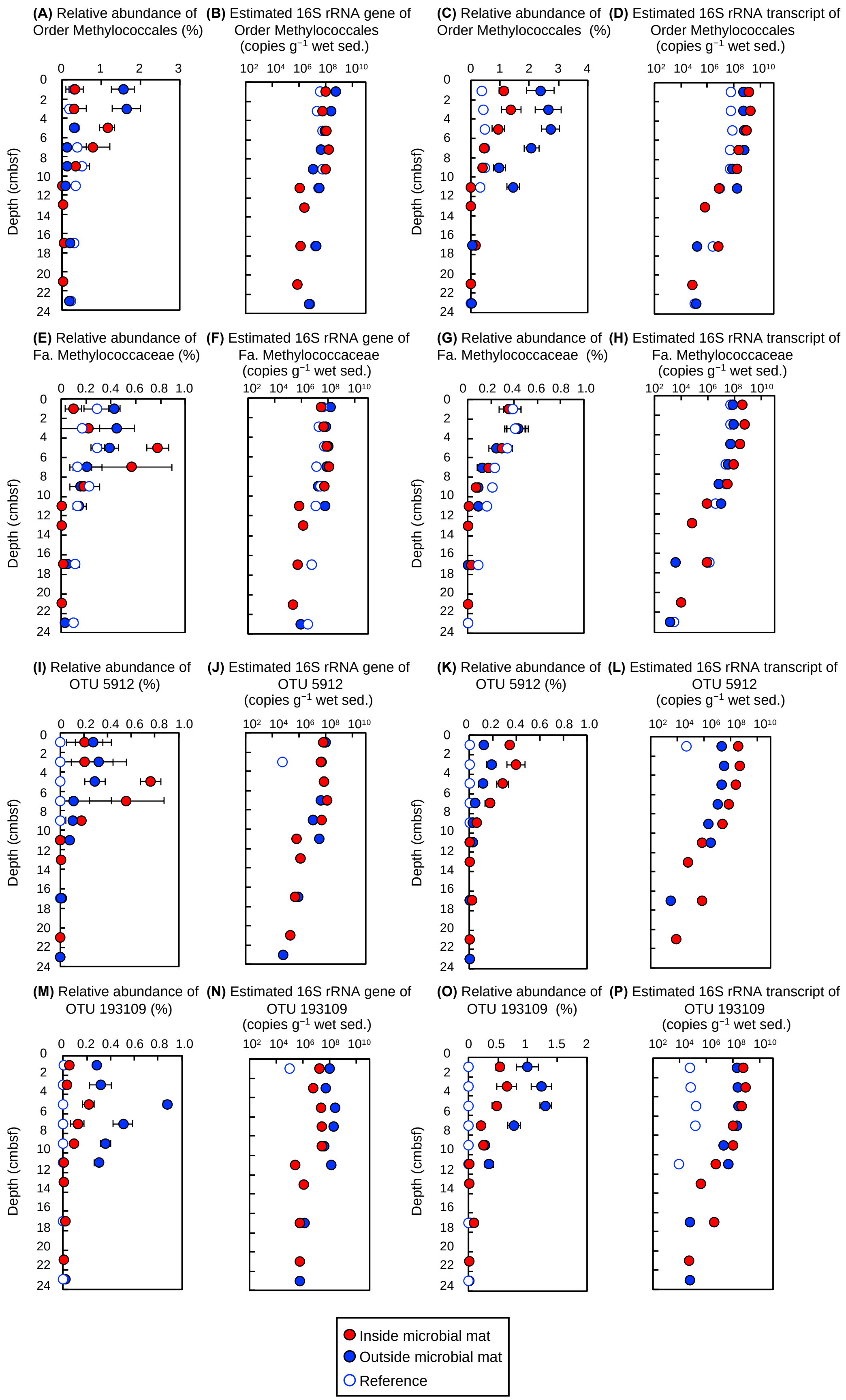
(A) Relative abundance of Order Methylococcales (%)
(B) Estimated 16S rRNA gene of Order Methylococcales (copies g^{-1} wet sed.)
(C) Relative abundance of Order Methylococcales (%)
(D) Estimated 16S rRNA transcript of Order Methylococcales (copies g^{-1} wet sed.)
(E) Relative abundance of Fa. Methylococcaceae (%)
(F) Estimated 16S rRNA gene of Fa. Methylococcaceae (copies g^{-1} wet sed.)
(G) Relative abundance of Fa. Methylococcaceae (%)
(H) Estimated 16S rRNA transcript of Fa. Methylococcaceae (copies g^{-1} wet sed.)
(I) Relative abundance of OTU 5912 (%)
(J) Estimated 16S rRNA gene of OTU 5912 (copies g^{-1} wet sed.)
(K) Relative abundance of OTU 5912 (%)
(L) Estimated 16S rRNA transcript of OTU 5912 (copies g^{-1} wet sed.)
(M) Relative abundance of OTU 193109 (%)
(N) Estimated 16S rRNA gene of OTU 193109 (copies g^{-1} wet sed.)
(O) Relative abundance of OTU 193109 (%)
(P) Estimated 16S rRNA transcript of OTU 193109 (copies g^{-1} wet sed.)
Depth (cmbsf)
Inside microbial mat
Outside microbial mat
Reference

Figure S7 Vertical distribution of putative aerobic methanotrophs in the sediments. (A–D) Methylococcales. (E–H) Methylococcaceae. (I–L) OTU 5912. (M–P) OTU 193109. Relative 16S rRNA gene (A, E, I, M) and transcript abundance (C, G, K, O) assessed by high-throughput sequencing (*n* = 3). The copy numbers of the 16S rRNA genes (B, F, J, N) and transcripts (D, H, L, P) were estimated by the relative abundance datasets and total copy numbers in Figure S6A, B.

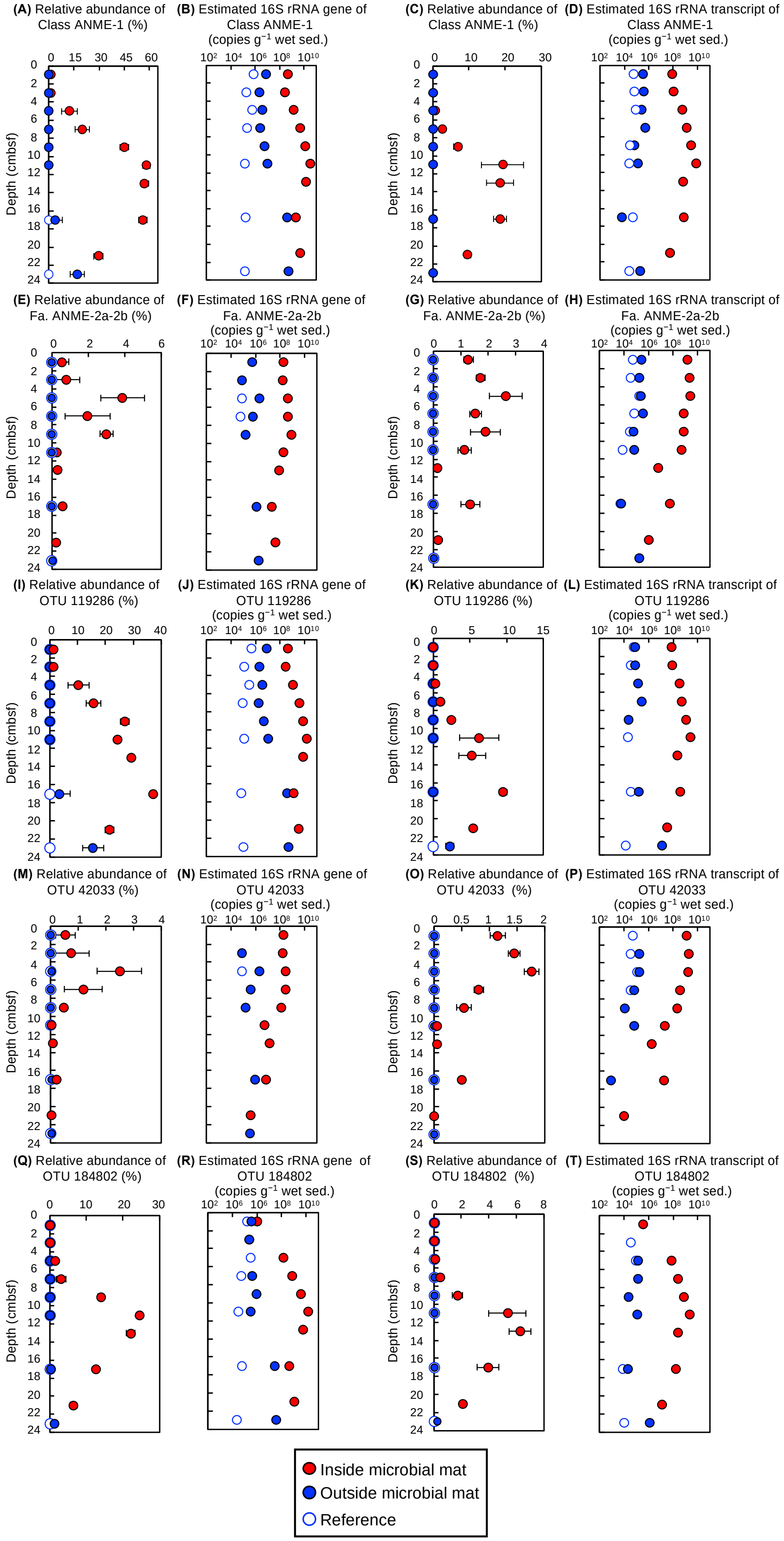
(A) Relative abundance of Class ANME-1 (%)
(B) Estimated 16S rRNA gene of Class ANME-1 (copies g−1 wet sed.)
(C) Relative abundance of Class ANME-1 (%)
(D) Estimated 16S rRNA transcript of Class ANME-1 (copies g−1 wet sed.)
(E) Relative abundance of Fa. ANME-2a-2b (%)
(F) Estimated 16S rRNA gene of Fa. ANME-2a-2b (copies g−1 wet sed.)
(G) Relative abundance of Fa. ANME-2a-2b (%)
(H) Estimated 16S rRNA transcript of Fa. ANME-2a-2b (copies g−1 wet sed.)
(I) Relative abundance of OTU 119286 (%)
(J) Estimated 16S rRNA gene of OTU 119286 (copies g−1 wet sed.)
(K) Relative abundance of OTU 119286 (%)
(L) Estimated 16S rRNA transcript of OTU 119286 (copies g−1 wet sed.)
(M) Relative abundance of OTU 42033 (%)
(N) Estimated 16S rRNA gene of OTU 42033 (copies g−1 wet sed.)
(O) Relative abundance of OTU 42033 (%)
(P) Estimated 16S rRNA transcript of OTU 42033 (copies g−1 wet sed.)
(Q) Relative abundance of OTU 184802 (%)
(R) Estimated 16S rRNA gene of OTU 184802 (copies g−1 wet sed.)
(S) Relative abundance of OTU 184802 (%)
(T) Estimated 16S rRNA transcript of OTU 184802 (copies g−1 wet sed.)
Depth (cmbsf)
Inside microbial mat
Outside microbial mat
Reference

Figure S8 Vertical distribution of putative anaerobic methanotrophs in the sediments. (A–D) ANME-1. (E–H) ANME-2a-2b. (I–L) OTU 119286. (M–P) OTU 42033. (Q–T) OTU 184802. Relative 16S rRNA gene (A, E, I, M, Q) and transcript abundance (C, G, K, O, S) assessed by high-throughput sequencing (n = 3). The copy numbers of the 16S rRNA genes (B, F, J, N, R) and transcripts (D, H, L, P, T) were estimated by the relative abundance datasets and total copy numbers in Figure S6A, B.

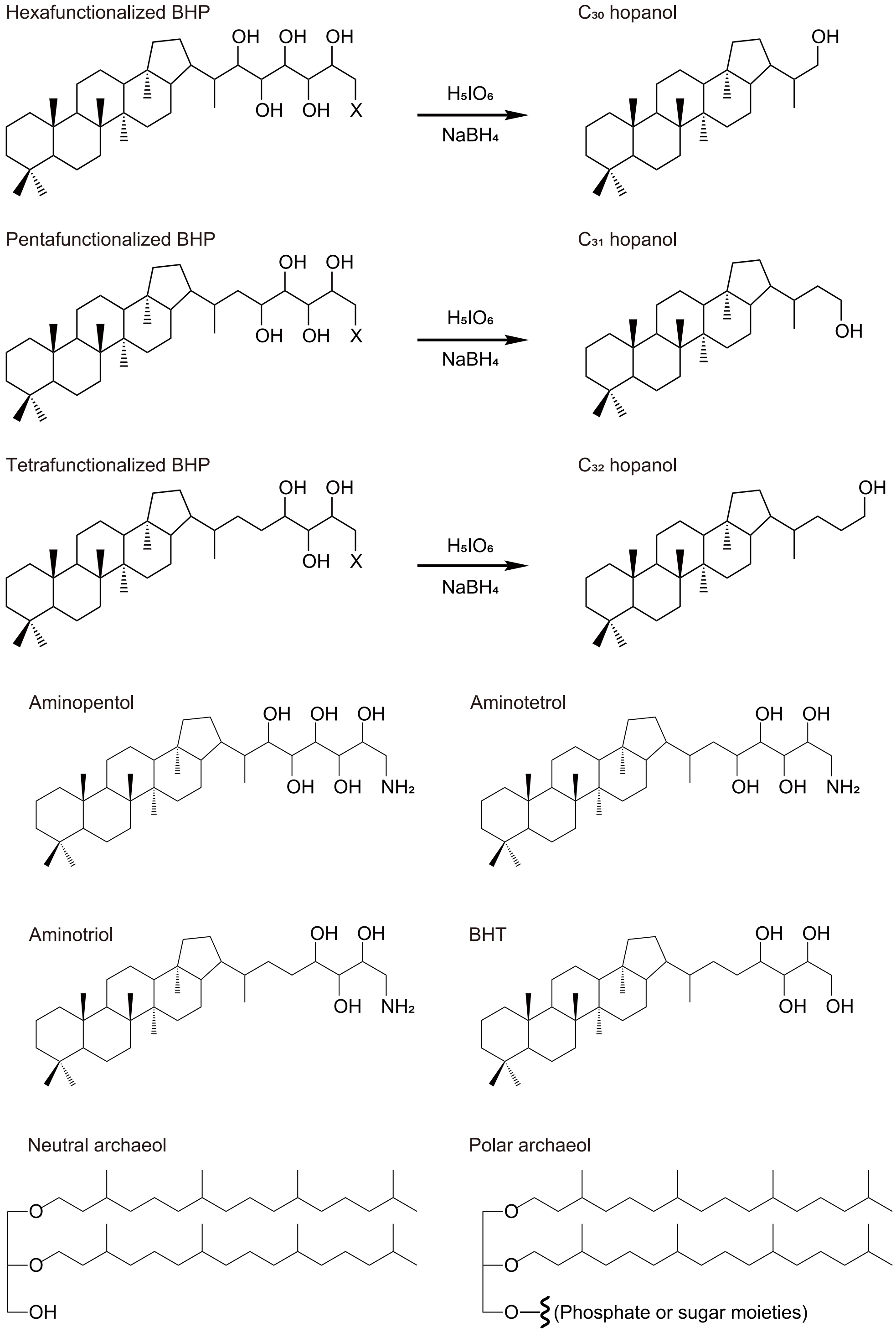

Figure S9 Structures of the bacterial and archaeal lipids. Upper panel shows periodic acid (H_5IO_6)/sodium borohydride ($NaBH_4$) reactants and products. BHP, bacteriohopanepolyol; BHT, bacteriohopanetetrol.

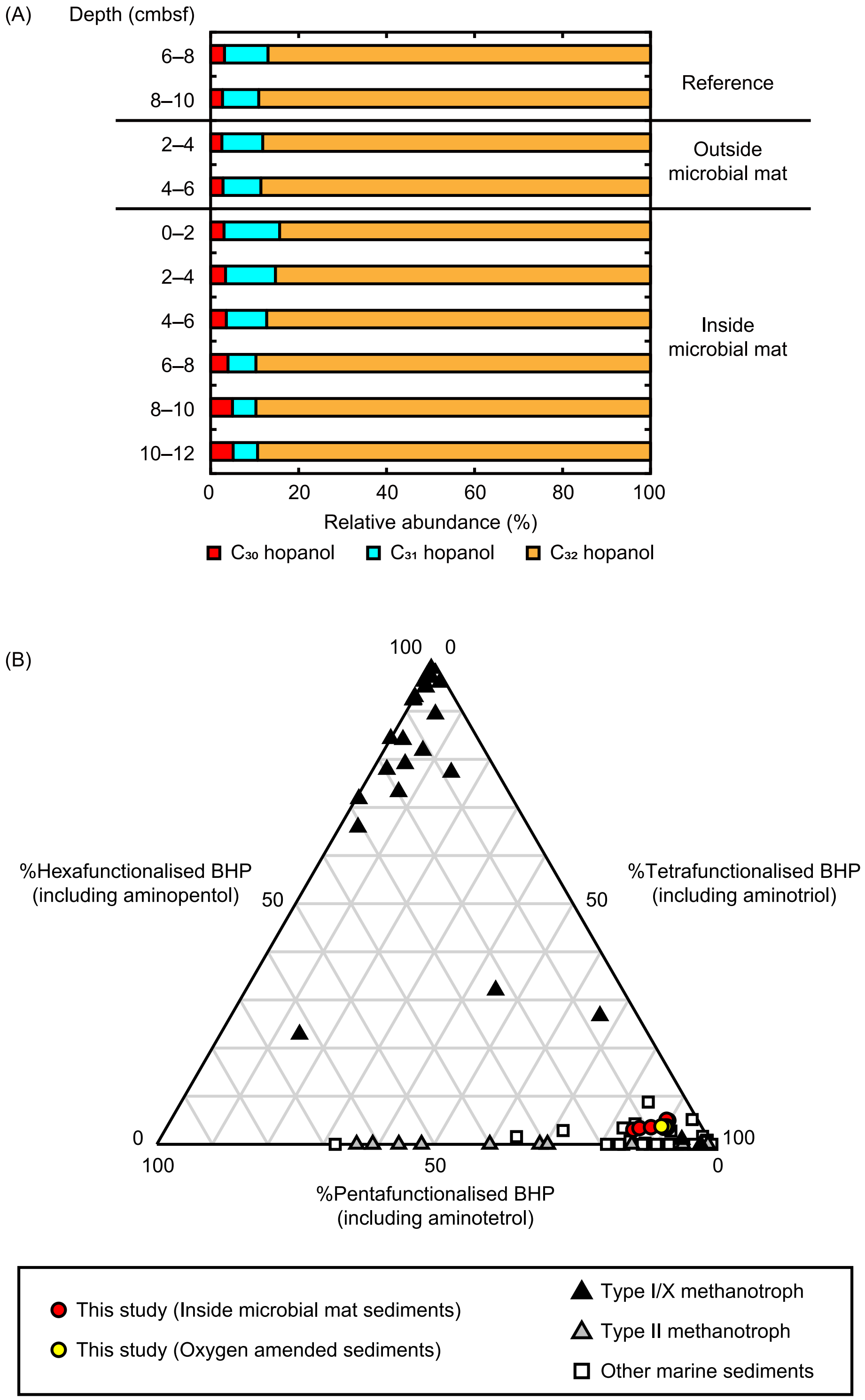
(A)
Depth (cmbsf)
6–8
8–10
2–4
4–6
0–2
2–4
4–6
6–8
8–10
10–12
Reference
Outside microbial mat
Inside microbial mat
0
20
40
60
80
100
Relative abundance (%)
C30 hopanol
C31 hopanol
C32 hopanol
(B)
100
0
%Hexafunctionalised BHP (including aminopentol)
%Tetrafunctionalised BHP (including aminotriol)
50
50
0
100
100
50
0
%Pentafunctionalised BHP (including aminotetrol)
This study (Inside microbial mat sediments)
This study (Oxygen amended sediments)
Type I/X methanotroph
Type II methanotroph
Other marine sediments

Figure S10 Relative abundance of hexa-, penta-, and tetra-functionalized BHPs in the sediments and cultured aerobic methanotrophic bacteria. (A) BHPs in the study site sediments. C_{30}, C_{31}, and C_{32} hopanols were derived from acid treatment of hexa-, penta-, and tetra-functionalized BHPs, respectively. (B) Comparison of BHPs in the inside microbial mat sediments with those of the cultured aerobic methanotrophs and other marine sediments. Sediments examined in this study are indicated as circles. Data for other marine sediments (squares) and cultured Type I/X and II methanotrophs (triangles) were sourced from previous studies [19, 20, 31, 32]. Note that BHPs plotted on this diagram include not only amino-BHPs (aminopentol, aminotetrol, and aminotriol) but also other hexa-, penta-, and tetra-functionalized BHPs and their unsaturated homologs. The relative abundance of BHPs examined in this study were determined by GC–MS, while data from the literature were determined by HPLC–MS. This figure was generated using R 4.1.1.

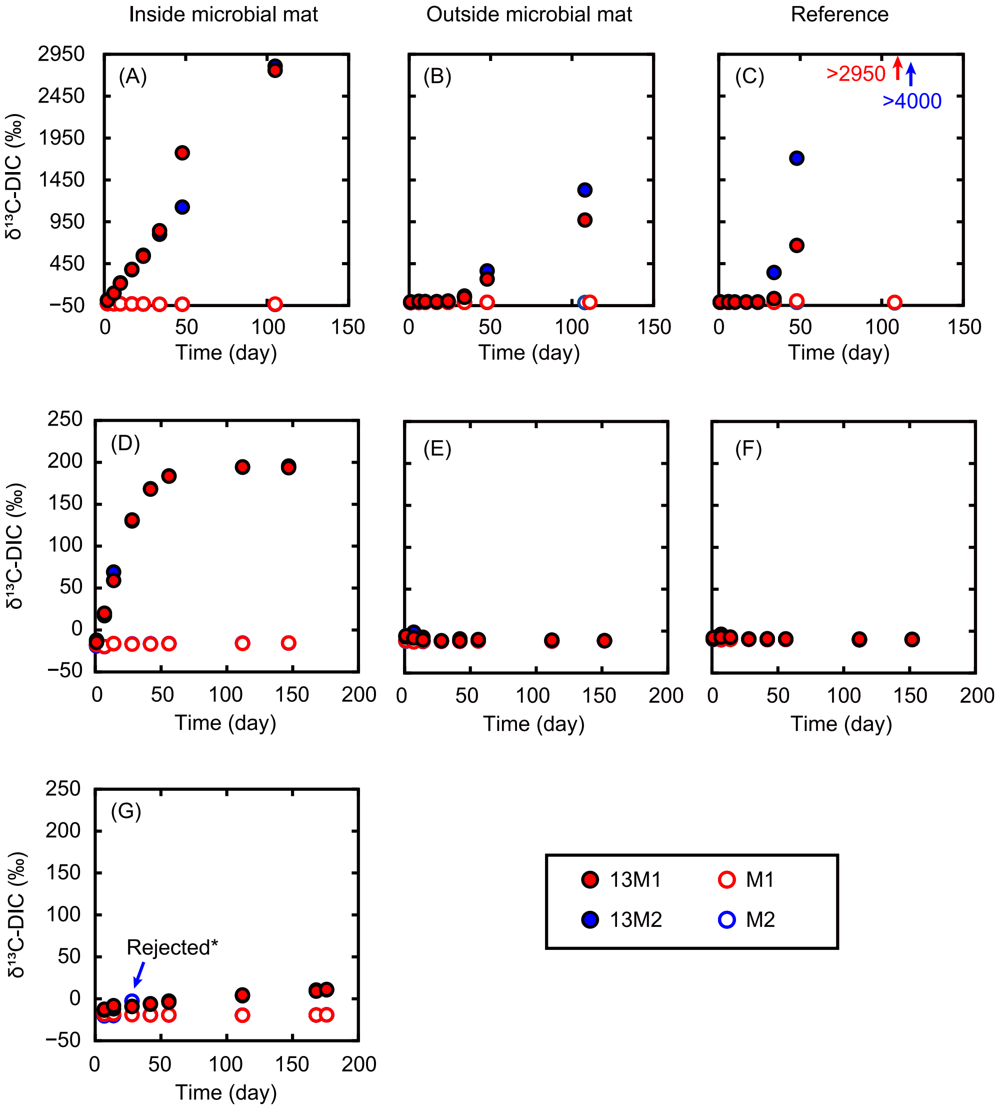

Figure S11 Temporal changes in ^{13}C abundance ($\delta^{13}C$ values) in DIC of sediments incubated with ^{13}C-labeled or non-labeled methane. (A–C) Oxygen amended incubation of the upper sediments in the inside microbial mat (A), outside microbial mat (B), and reference site (C). (D) Anaerobic incubation of the upper sediments in the inside microbial mat. (E–G) Anaerobic incubation of the lower sediments in the outside microbial mat (E), reference site (F), and inside microbial mat (G). All incubations were performed in duplicate for both ^{13}C-labeled methane (13M1 and 13M2, closed circles) and non-labeled methane treatments (M1 and M2, open circles). *Anomalously high $\delta^{13}C$ value even without ^{13}C-methane (M2) amendment for determination of the methane oxidation rate.

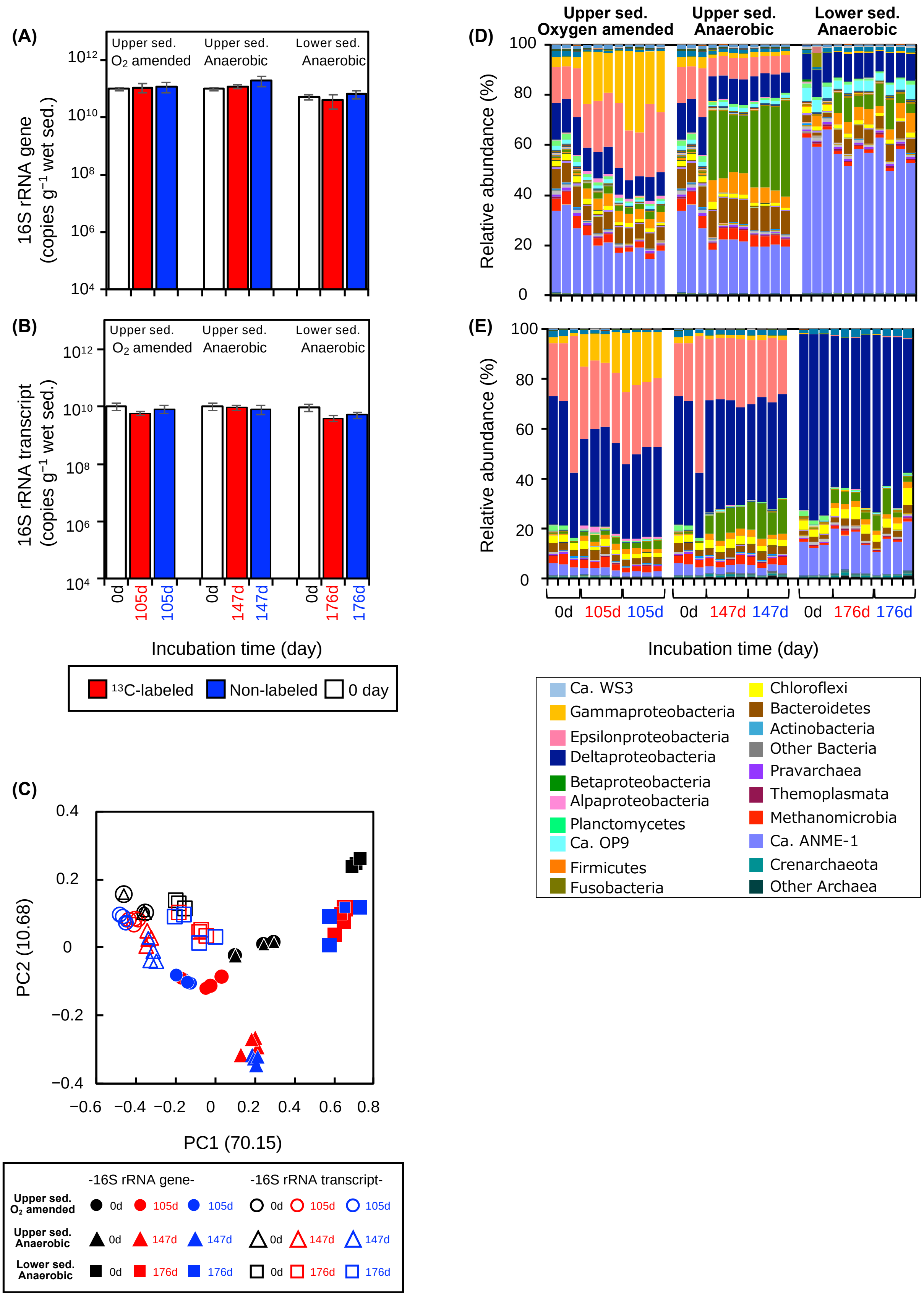

(A)
Upper sed. O2 amended
Upper sed. Anaerobic
Lower sed. Anaerobic
16S rRNA gene (copies g−1 wet sed.)
(B)
16S rRNA transcript (copies g−1 wet sed.)
0d 105d 105d 0d 147d 147d 0d 176d 176d
Incubation time (day)
13C-labeled Non-labeled 0 day
(C)
PC2 (10.68)
PC1 (70.15)
-16S rRNA gene-
-16S rRNA transcript-
(D)
Upper sed. Oxygen amended
Upper sed. Anaerobic
Lower sed. Anaerobic
Relative abundance (%)
(E)
Ca. WS3
Gammaproteobacteria
Epsilonproteobacteria
Deltaproteobacteria
Betaproteobacteria
Alpaproteobacteria
Planctomycetes
Ca. OP9
Firmicutes
Fusobacteria
Chloroflexi
Bacteroidetes
Actinobacteria
Other Bacteria
Pravarchaea
Themoplasmata
Methanomicrobia
Ca. ANME-1
Crenarchaeota
Other Archaea

Figure S12 Temporal changes in 16S rRNA gene- and transcript-based microbial community compositions during ^{13}C-tracer incubation of the inside microbial mat sediments. (A, B) Copy numbers assessed by quantitative PCR (A) and RT-PCR (B) (*n* = 3 or 4). (C) PCoA plot of high-throughput sequence data (*n* = 3 or 4) based on the equal numbers (*n* = 31,349) of sequences. The incubation times are indicated by the different colors. (D, E) Phylogenetic assignment at the phylum and class levels (in different colors) for the 16S rRNA gene (D) and transcript sequences (E) generated using QIIME software version 1.7.051 with the Greengenes 16S rRNA database version 13_8. Detailed information of the determined sequence is shown in Table S5.

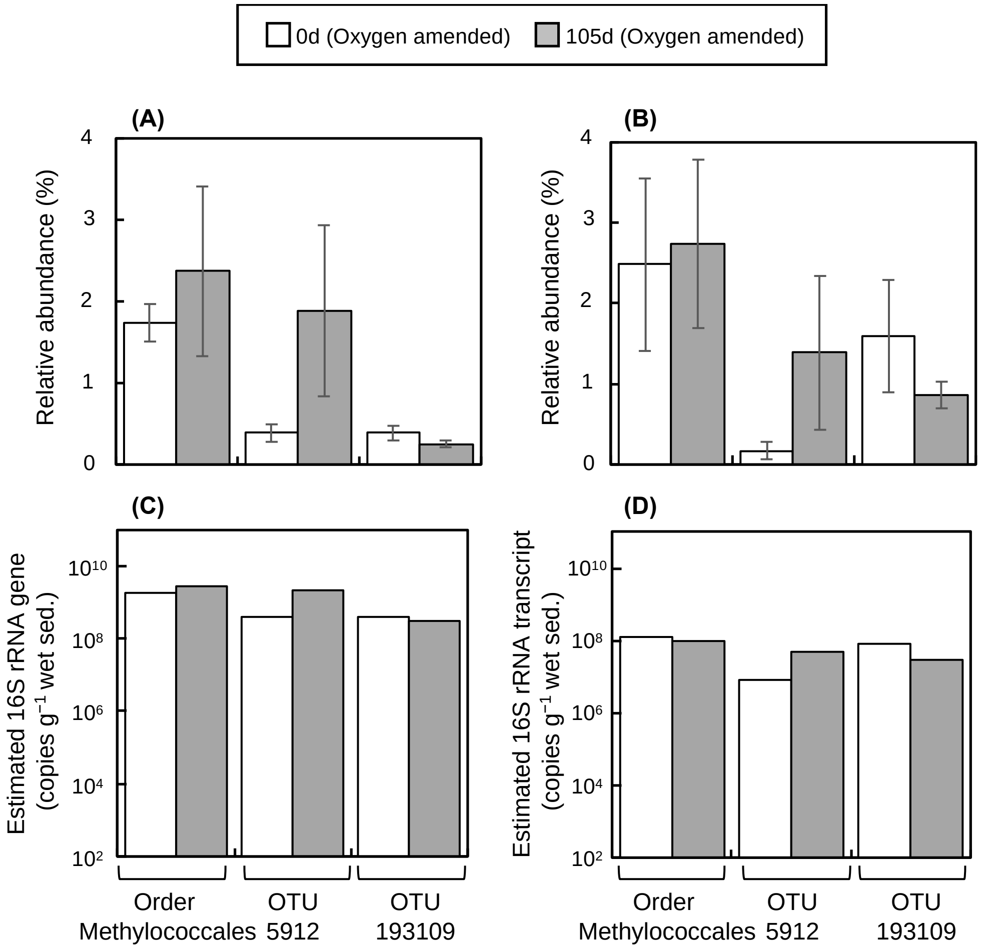

Figure S13 Temporal changes in specific aerobic methanotrophs (Methylococcales, OTUs 5912 and 193109) during ^{13}C-tracer incubation of the upper sediments of the inside microbial mat. (A, B) Relative 16S rRNA gene (A) and transcript abundance (B) assessed by high-throughput sequencing at the initial (*n* = 3) and final (*n* = 8). (C, D) 16S rRNA gene (C) and transcript copy numbers (D) estimated by the relative abundance datasets and total copy numbers in Figure S12A, B.

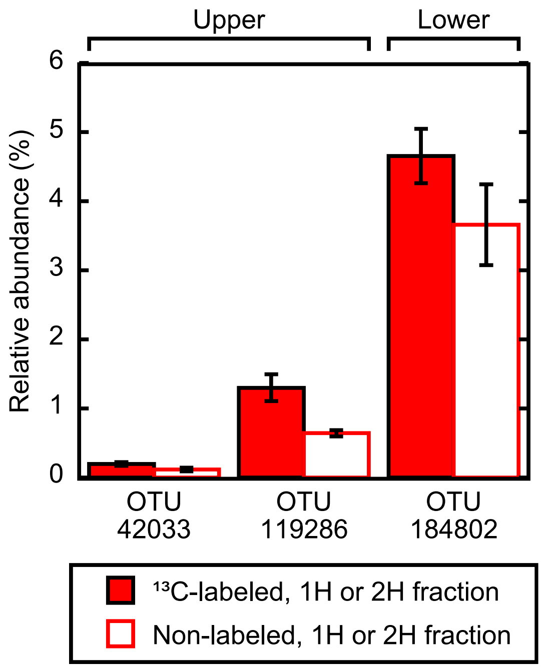

Figure S14 Relative abundance of 16S rRNA transcripts from OTUs 119286 and 184802 (ANME-1a) and OTU 42033 (ANME-2a-2b) in the density-resolved heavy fractions after anaerobic ^{13}C-tracer incubation of the upper and lower sediments of the inside microbial mat. The higher abundances found in the labeled incubation than in the non-labeled incubation (both $n = 3$) indicate the incorporation of ^{13}C-labeled methane into the 16S rRNA transcripts. Error bars indicate standard deviation. "1H," first-heaviest fraction; "2H," second-heaviest fraction.

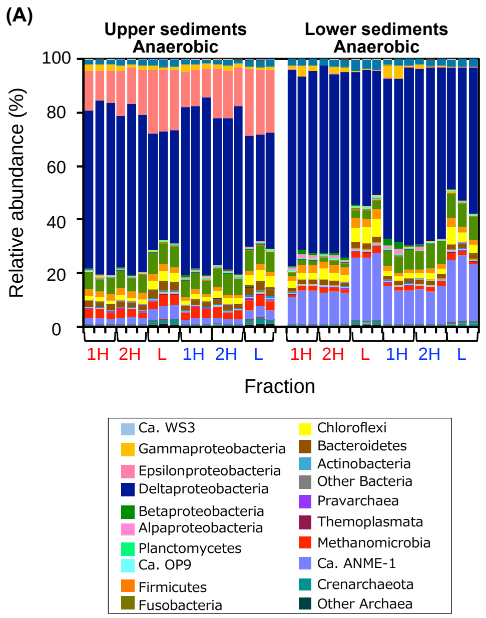

(B) 1H vs 1H (Upper sediments, Anaerobic)

OTU	Closest relative	Identity	Acc. No.
21157	*Desulfatibacillum alkenivorans*	94.1%	NR025795
36467	*Ilyobacter delafieldii*	98.0%	NR104743
24462	*Ancylomarina subtilis*	98.8%	NR152047
12751	*Clostridium grantii*	99.6%	NR026131
33191	*Fusibacter fontis*	93.7%	NR137378
14686	*Vallitalea pronyensis*	90.1%	NR125677
1074	*Desulfatibacillum alkenivorans*	96.1%	NR025795
25672	*Ilyobacter polytropus*	98.4%	NR074506
10517	*Tepidibacter mesophilus*	83.9%	NR108539
8978	*Sulfurimonas gotlandica*	93.3%	NR121690
24453	*Arcobacter pacificus*	94.5%	NR148627
3517	*Desulfofaba fastidiosa*	96.9%	NR025746

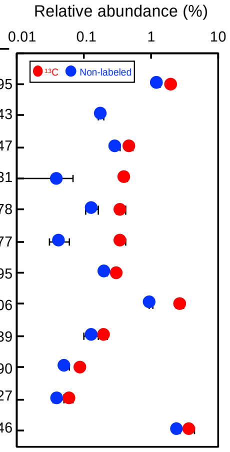

(C) 2H vs 2H (Upper sediments, Anaerobic)

OTU	Closest relative	Identity	Acc. No.
14686	*Vallitalea pronyensis*	90.1%	NR125677
36467	*Ilyobacter delafieldii*	98.0%	NR104743
33191	*Fusibacter fontis*	93.7%	NR137378
35103	*Oceanispirochaeta sediminicola*	95.3%	NR158023
**42033**	**Uncultured ANME-2a-2b**	**99.2%**	**FJ555678**
12751	*Clostridium grantii*	99.6%	NR026131
41612	*Thioprofundum hispidum*	94.9%	NR112620
24462	*Ancylomarina subtilis*	98.8%	NR152047
25672	*Ilyobacter polytropus*	98.4%	NR074506
**119286**	**Uncultured ANME-1a**	**97.2%**	**AF134392**
1074	*Desulfatibacillum alkenivorans*	96.1%	NR025795
41558	*Thermomarinilinea lacunifontana*	89.8%	NR132293
6649	*Rubrivirga marina*	84.6%	NR109654

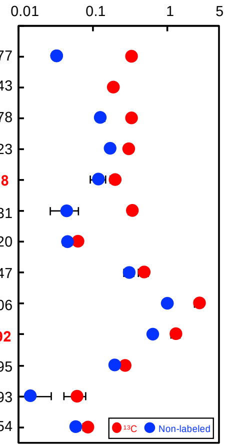

(D) 1H vs 1H (Lower sediments, Anaerobic)

OTU	Closest relative	Identity	Acc. No.
31676	*Pseudoramibacter alactolyticus*	82.0%	NR109529
12560	*Thermomarinilinea lacunifontana*	87.8%	NR132293
39172	*Marispirochaeta aestuarii*	92.9%	NR158116
**184802**	**Uncultured ANME-1a**	**98.0%**	**AY714817**
33421	*Desulfomonile tiedjei*	89.4%	NR074118

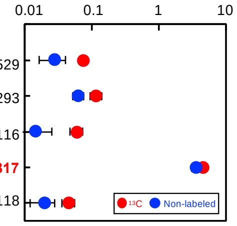

(E) 2H vs 2H (Lower sediments, Anaerobic)

OTU	Closest relative	Identity	Acc. No.
16311	*Alkaliphilus metalliredigens*	92.5%	NR074633
27844	*Thermomarinilinea lacunifontana*	84.0%	NR132293
7755	*Marinisporobacter balticus*	100%	NR156975
10384	*Desulfuromonas acetoxidans*	87.4%	NR121678
25236	*Exiguobacterium aurantiacum*	100%	NR113666

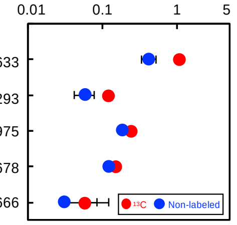

Figure S15 Microbial community composition of density-resolved fractions based on high-sensitivity RNA-stable isotope probing (SIP). (A) Phylogenetic assignment at the phylum and class levels (in different colors) generated using QIIME software version 1.7.051 with the Greengenes 16S rRNA database version 13_8. Detailed information of the determined sequence and buoyant density is shown in Table S6. (B–E) Closest relatives (species name, sequence similarity, and accession no.) of the significant ^{13}C-incorporating OTUs with >1.2-fold greater relative abundances in the ^{13}C treatments than those in the non-labeled treatments ($P < 0.05$, $n = 3$). Known ANME species are indicated in red.

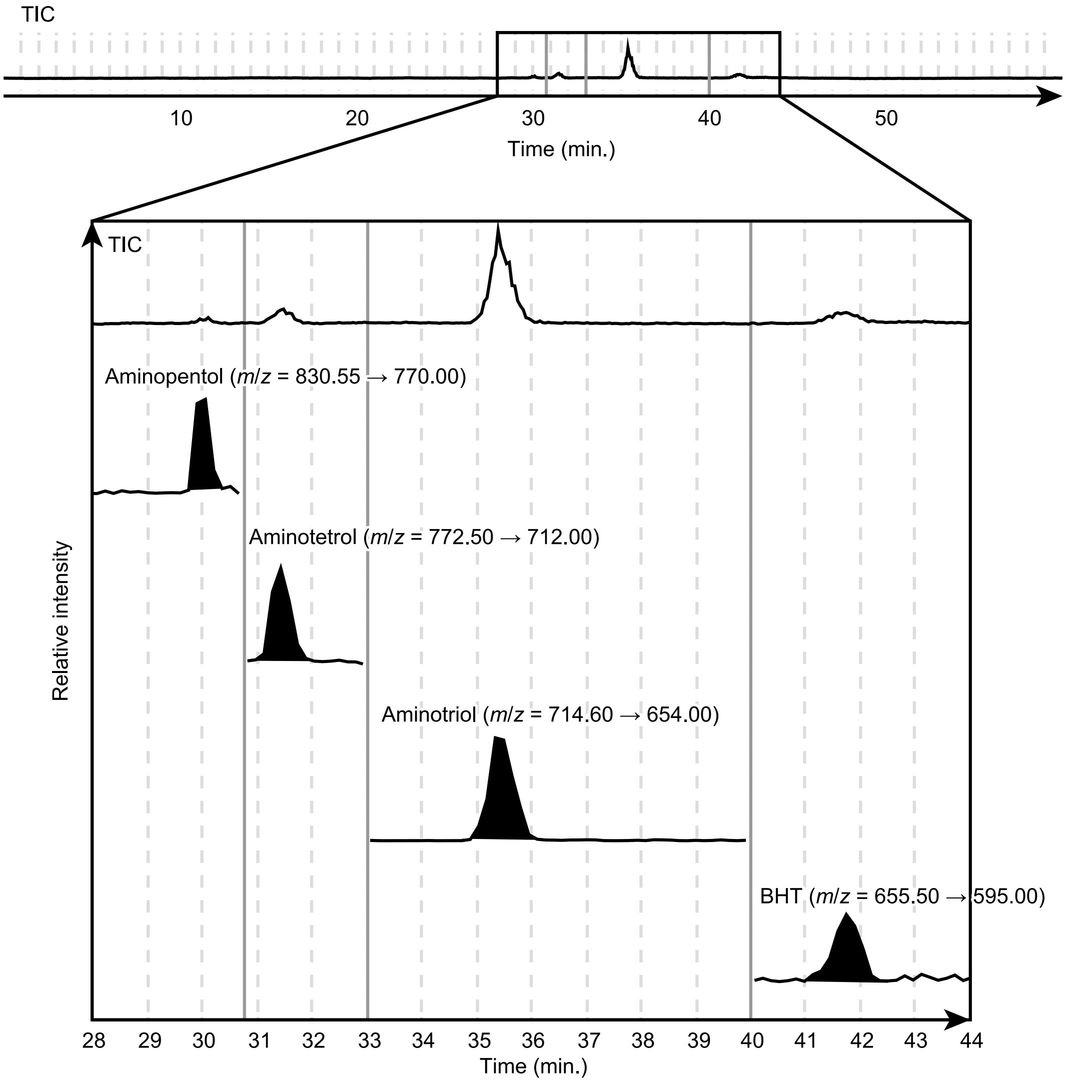

Figure S16 Total and extracted ion chromatograms (TIC and EIC, respectively) obtained by HPLC–MS analysis of oxygen amended sediments in product ion monitoring mode. Product ions were monitored for precursor ions of *m*/*z* 830.55 (acetylated aminopentol, 0–30.75 min), 772.50 (acetylated aminotetrol, 30.75–33 min), 714.60 (acetylated aminotriol, 33–40 min), and 655.50 (acetylated BHT, 40–60 min). EICs were extracted for product ions resulting from loss of one functional group as acetic acid (*m*/*z* 60). Scaled to the largest peak in each chromatogram. BHT, bacteriohopanetetrol.

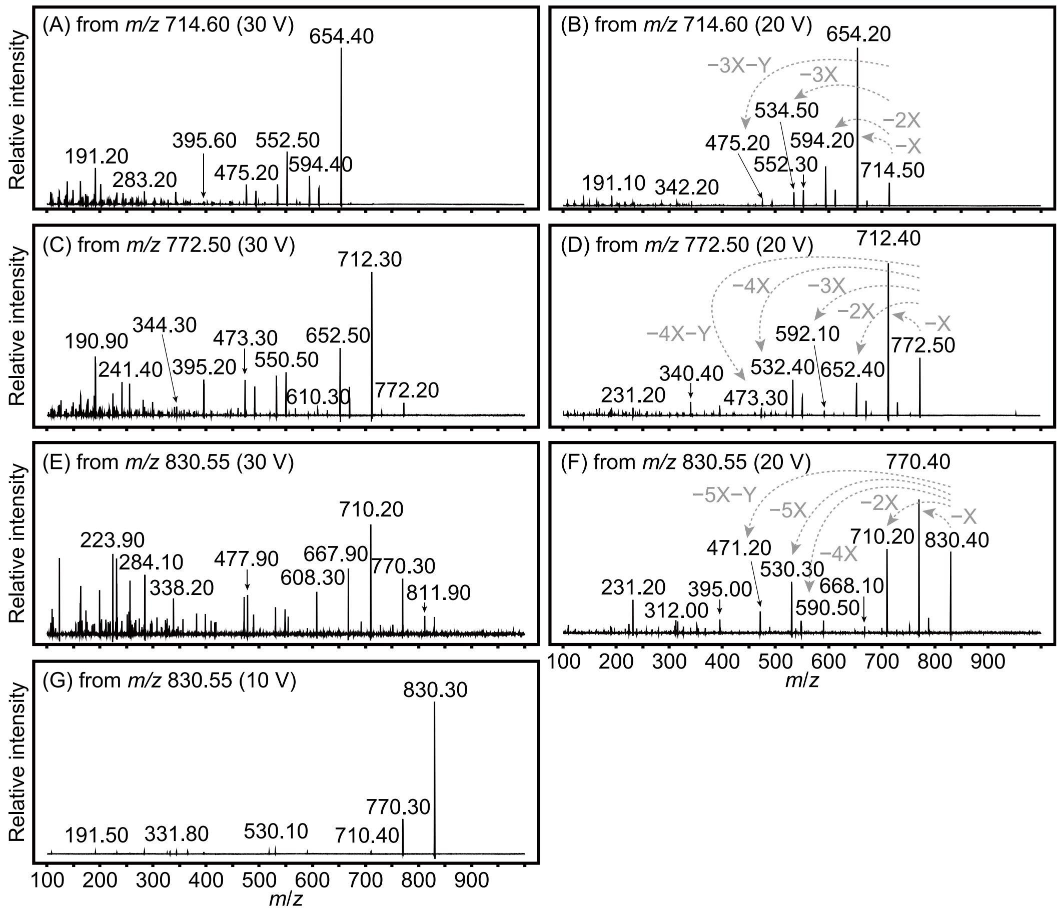

Figure S17 Mass spectra of amino-BHPs in oxygen amended sediments obtained in product ion monitoring mode. (A, B) Aminotriol. (C, D) Aminotetrol. (E–G) Aminopentol. Precursor ion and collision energy (in parentheses) are shown at the upper left of each panel. Fragmentation patterns are indicated by gray dashed arrows: X = CH_3COOH (60 Da); Y = CH_3CONH_2 (59 Da).

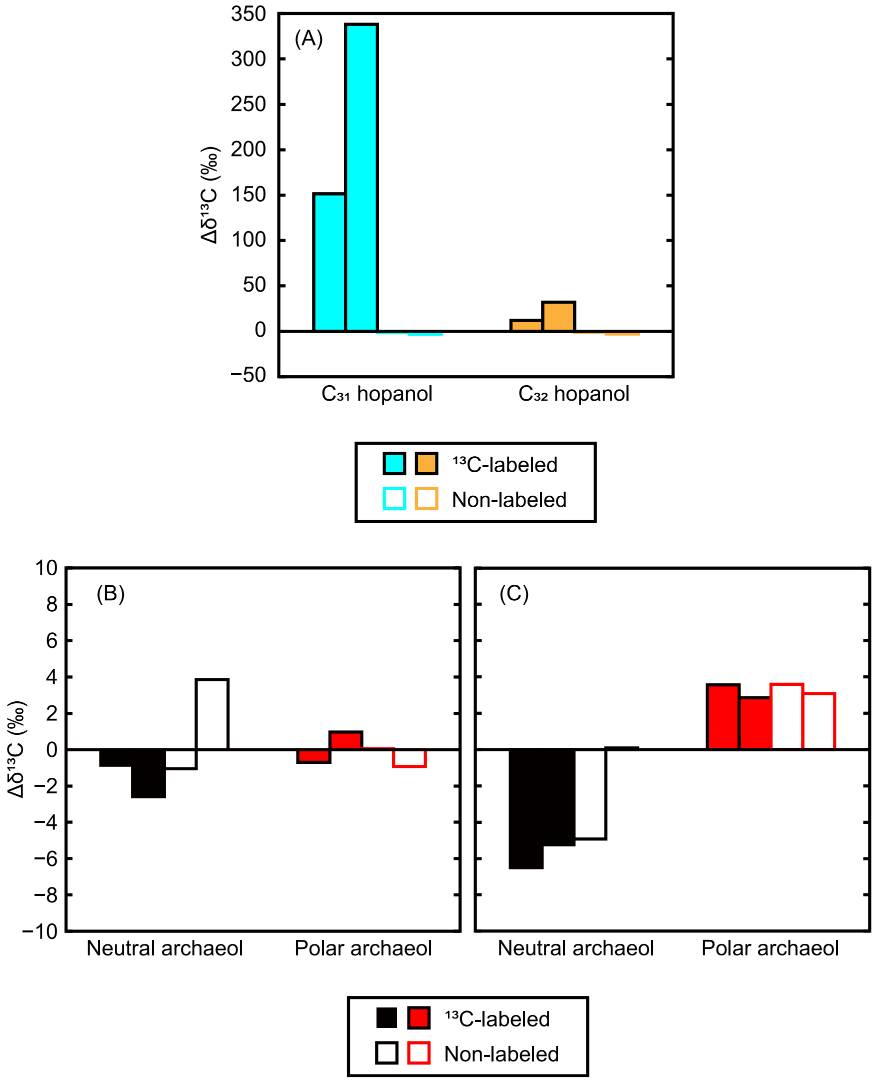

Figure S18 Changes in δ^{13}C values of lipids after ^{13}C-tracer incubation of the inside microbial mat sediments. (A) C_{31} and C_{32} hopanols derived from penta- and tetra-functionalized BHPs, respectively, in oxygen amended incubation of the upper sediments. (B, C) Neutral and polar archaeols in anaerobic incubation of the upper (B) and lower (C) sediments. Shown as differences from the initial values at day 0 (Δδ^{13}C values, *n* = 2 each for the labeled and non-labeled incubations).

SI References

(1) Okamura, Y.; Watanabe, M.; Morijiri, R.; Satoh, M. Rifting and basin inversion in the eastern margin of the Japan Sea. *Island Arc* **1995**, *4*, 166–181.
(2) Monzawa, N.; Kaneko, M.; Osawa, M. A review of petroleum system in the deep water area of the Toyama Trough to the Sado Island in the Japan Sea, based on the results of the METI Sado Nansei Oki drilling. *J. Japanese Assoc. Petrol. Technol.* **2006**, *71*, 618–627.
(3) Okui, A.; Kaneko, M.; Nakanishi, S.; Monzawa, N.; Yamamoto, H. An integrated approach to understanding the petroleum system of a frontier deep-water area, offshore Japan. *Petrol. Geosci.* **2008**, *14*, 223–233.
(4) Matsumoto, R.; Okuda, Y.; Hiruta, A.; Tomaru, H.; Takeuchi, E.; Sanno, R.; Suzuki, M.; Tsuchinaga, K.; Ishida, Y.; Ishizaki, O.; Takeuchi, R.; Komatsubara, J.; Freire, A. F.; Machiyama, H.; Aoyama, C.; Joshima, M.; Hiromatsu, M.; Snyder, G.; Numanami, H.; Satoh, M.; Matoba, Y.; Nakagawa, H.; Kakuwa, Y.; Ogihara, S.; Yanagawa, K.; Sunamura, M.; Goto, T.; Lu, H.; Kobayashi, T. Formation and collapse of gas hydrate deposits in high methane flux area of the Joetsu Basin, eastern margin of Japan Sea. *J. Geogr.* **2009**, *118*, 43–71.
(5) Matsumoto, R.; Tanahashi, M.; Kakuwa, Y.; Snyder, G.; Ohkawa, S.; Tomaru, H.; Morita, S. Recovery of thick deposits of massive gas hydrates from gas chimney structures, eastern margin of Japan Sea: Japan Sea Shallow Gas Hydrate Project. *Fire in the Ice* **2017**, *17*, 1–6.
(6) Whiticar, M. J. Carbon and hydrogen isotope systematics of bacterial formation and oxidation of methane. *Chem. Geol.* **1999**, *161*, 291–314.
(7) Ota, Y.; Suzumura, M.; Tsukasaki, A.; Suzuki, A.; Yamaoka, K.; Asada, M.; Satoh, M. Anaerobic oxidation of methane and trace-element geochemistry in microbial mat-covered sediments related to methane seepage, northeastern Japan Sea. *Chem. Geol.* **2022**, *611*, 121093.
(8) Kinoshita, M.; Goto, S.; Yamano, M. Estimation of thermal gradient and diffusivity by means of long-term measurements of subbottom temperatures at western Sagami Bay, Japan. *Earth Planet. Sci. Lett.* **1996**, *141*, 249–258.
(9) Davis, E. E.; Wang, K. L.; Becker, K.; Thomson, R. E.; Yashayaev, I. Deep-ocean temperature variations and implications for errors in seafloor heat flow determinations. *J. Geophys. Res. Solid Earth* **2003**, *108*, 2034.
(10) Hamamoto, H.; Yamano, M.; Goto, S. Heat flow measurement in shallow seas through long-term temperature monitoring. *Geophys. Res. Lett.* **2005**, *32*, L21311.
(11) Goto, S.; Yamano, M.; Kinoshita, M. Thermal response of sediment with vertical fluid flow to periodic temperature variation at the surface. *J. Geophys. Res. Solid Earth* **2005**, *110*, B01106.
(12) Tarantola, A. *Inverse Problem Theory and Methods for Model Parameter Estimation*; Society for Industrial and Applied Mathematics: Philadelphia, USA, 2005.
(13) Kaye, G. W. C.; Laby, T. H. *Tables of Physical and Chemical Constants*; Longmans: Essex, England, 1995.
(14) Von Herzen, R.; Maxwell, A. E. The measurement of thermal conductivity of deep-sea sediments by a needle-probe method. *J. Geophys. Res.* **1959**, *64*, 1557–1563.

(15) Aoyagi, T.; Hanada, S.; Itoh, H.; Sato, Y.; Ogata, A.; Friedrich, M. W.; Kikuchi, Y.; Hori, T. Ultra-high-sensitivity stable-isotope probing of rRNA by high-throughput sequencing of isopycnic centrifugation gradients. *Environ. Microbiol. Rep.* **2015**, *7*, 282–287.
(16) Aoyagi, T.; Inaba, T.; Aizawa, H.; Mayumi, D.; Sakata, S.; Charfi, A.; Suh, C.; Lee, J. H.; Sato, Y.; Ogata, A.; Habe, H.; Hori, T. Unexpected diversity of acetate degraders in anaerobic membrane bioreactor treating organic solid waste revealed by high-sensitivity stable isotope probing. *Water Res.* **2020**, *176*, 115750.
(17) Caporaso, J. G.; Kuczynski, J.; Stombaugh, J.; Bittinger, K.; Bushman, F. D.; Costello, E. K.; Fierer, N.; Pena, A. G.; Goodrich, J. K.; Gordon, J. I.; Huttley, G. A.; Kelley, S. T.; Knights, D.; Koenig, J. E.; Ley, R. E.; Lozupone, C. A.; McDonald, D.; Muegge, B. D.; Pirrung, M.; Reeder, J.; Sevinsky, J. R.; Tumbaugh, P. J.; Walters, W. A.; Widmann, J.; Yatsunenko, T.; Zaneveld, J.; Knight, R. QIIME allows analysis of high-throughput community sequencing data. *Nat. Methods* **2010**, *7*, 335–336.
(18) Oba, M.; Sakata, S.; Tsunogai, U. Polar and neutral isopranyl glycerol ether lipids as biomarkers of archaea in near-surface sediments from the Nankai Trough. *Org. Geochem.* **2006**, *37*, 1643–1654.
(19) Rohmer, M.; Bouviernave, P.; Ourisson, G. Distribution of hopanoid triterpenes in prokaryotes. *J. Gen. Microbiol.* **1984**, *130*, 1137–1150.
(20) van Winden, J. F.; Talbot, H. M.; Kip, N.; Reichart, G.-J.; Pol, A.; McNamara, N. P.; Jetten, M. S. M.; Op den Camp, H. J. M.; Sinninghe Damsté, J. S. Bacteriohopanepolyol signatures as markers for methanotrophic bacteria in peat moss. *Geochim. Cosmochim. Acta* **2012**, *77*, 52–61.
(21) Talbot, H. M.; Squier, A. H.; Keely, B. J.; Farrimond, P. Atmospheric pressure chemical ionisation reversed-phase liquid chromatography/ion trap mass spectrometry of intact bacteriohopanepolyols. *Rapid Commun. Mass Spectrom.* **2003**, *17*, 728–737.
(22) Welander, P. V.; Summons, R. E. Discovery, taxonomic distribution, and phenotypic characterization of a gene required for 3-methylhopanoid production. *Proc. Natl. Acad. Sci. USA.* **2012**, *109*, 12905–12910.
(23) Kusch, S.; Shah Walter, S. R.; Hemingway, J. D.; Pearson, A. Improved chromatography reveals multiple new bacteriohopanepolyol isomers in marine sediments. *Org. Geochem.* **2018**, *124*, 12–21.
(24) Katayama, T.; Yoshioka, H.; Takahashi, H. A.; Amo, M.; Fujii, T.; Sakata, S. Changes in microbial communities associated with gas hydrates in subseafloor sediments from the Nankai Trough. *FEMS Microbiol. Ecol.* **2016**, *92*, fiw093.
(25) Marlow, J. J.; Hoer, D.; Jungbluth, S. P.; Reynard, L. M.; Gartman, A.; Chavez, M. S.; El-Naggar, M. Y.; Tuross, N.; Orphan, V. J.; Girguis, P. R. Carbonate-hosted microbial communities are prolific and pervasive methane oxidizers at geologically diverse marine methane seep sites. *Proc. Natl. Acad. Sci. USA.* **2021**, *118*, e2006857118.
(26) Aoyagi, T.; Morishita, F.; Sugiyama, Y.; Ichikawa, D.; Mayumi, D.; Kikuchi, Y.; Ogata, A.; Muraoka, K.; Habe, H.; Hori, T. Identification of active and taxonomically diverse 1,4-dioxane degraders in a full-scale activated sludge system by high-sensitivity stable isotope probing. *ISME J.* **2018**, *12*, 2376–2388.
(27) Lueders, T.; Manefield, M.; Friedrich, M. W. Enhanced sensitivity of DNA- and rRNA-based stable isotope probing by fractionation and quantitative analysis of isopycnic centrifugation gradients. *Environ. Microbiol.* **2004**, *6*, 73–78.

(28) Quast, C.; Pruesse, E.; Yilmaz, P.; Gerken, J.; Schweer, T.; Yarza, P.; Peplies, J.; Glockner, F. O. The SILVA ribosomal RNA gene database project: Improved data processing and web-based tools. *Nucleic Acids Res.* **2013**, *41*, D590–D596.
(29) Pruesse, E.; Peplies, J.; Glockner, F. O. SINA: Accurate high-throughput multiple sequence alignment of ribosomal RNA genes. *Bioinformatics* **2012**, *28*, 1823–1829.
(30) Asada, M.; Satoh, M.; Tanahashi, M.; Yokota, T.; Goto, S. Visualization of shallow subseafloor fluid migration in a shallow gas hydrate field using high-resolution acoustic mapping and ground-truthing and their implications on the formation process: A case study of the Sakata Knoll on the eastern margin of the Sea of Japan. *Mar. Geophys. Res.* **2022**, *43*, 34.
(31) Rush, D.; Osborne, K. A.; Birgel, D.; Kappler, A.; Hirayama, H.; Peckmann, J.; Poulton, S. W.; Nickel, J. C.; Mangelsdorf, K.; Kalyuzhnaya, M.; Sidgwick, F. R.; Talbot, H. M. The bacteriohopanepolyol inventory of novel aerobic methane oxidising bacteria reveals new biomarker signatures of aerobic methanotrophy in marine systems. *PLoS One* **2016**, *11*, e0165635.
(32) Jahnke, L. L.; Summons, R. E.; Hope, J. M.; des Marais, D. J. Carbon isotopic fractionation in lipids from methanotrophic bacteria II: The effects of physiology and environmental parameters on the biosynthesis and isotopic signatures of biomarkers. *Geochim. Cosmochim. Acta* **1999**, *63*, 79–93.
